# Orbit/CLASP determines centriole length by antagonising Klp10A in *Drosophila* spermatocytes

**DOI:** 10.1242/jcs.251231

**Published:** 2021-03-26

**Authors:** Tsuyoshi Shoda, Kanta Yamazoe, Yuri Tanaka, Yuki Asano, Yoshihiro H. Inoue

**Affiliations:** Department of Insect Biomedical Research, Centre for Advanced Insect Research Promotion, Kyoto Institute of Technology, Kyoto 606-8585, Japan

**Keywords:** Centriole elongation, *Drosophila*, Spermatocyte, Orbit, CLASP, Klp10A, Cp110

## Abstract

After centrosome duplication, centrioles elongate before M phase. To identify genes required for this process and to understand the regulatory mechanism, we investigated the centrioles in *Drosophila* premeiotic spermatocytes expressing fluorescently tagged centriolar proteins. We demonstrated that an essential microtubule polymerisation factor, Orbit (the *Drosophila* CLASP orthologue, encoded by *chb*), accumulated at the distal end of centrioles and was required for the elongation. Conversely, a microtubule-severing factor, Klp10A, shortened the centrioles. Genetic analyses revealed that these two proteins functioned antagonistically to determine centriole length. Furthermore, Cp110 in the distal tip complex was closely associated with the factors involved in centriolar dynamics at the distal end. We observed loss of centriole integrity, including fragmentation of centrioles and earlier separation of the centriole pairs, in *Cp110*-null mutant cells either overexpressing Orbit or depleted of *Klp10A*. Excess centriole elongation in the absence of the distal tip complex resulted in the loss of centriole integrity, leading to the formation of multipolar spindle microtubules emanating from centriole fragments, even when they were unpaired. Our findings contribute to understanding the mechanism of centriole integrity, disruption of which leads to chromosome instability in cancer cells.

## Abstract (precis)

**Summary:** A microtubule polymerising factor, Orbit, localises on centrioles and determines centriole length by antagonising Klp10A in *Drosophila* spermatocytes.

## INTRODUCTION

The centrosome plays an indispensable role as the major microtubule-organising centre (MTOC) in a cell. During mitosis, centrosomes duplicated in the S phase move apart from each other and reach the opposite poles of the cell. Each centrosome is involved in the assembly of spindle poles, which enables construction of the bipolar spindle microtubule structure (Vitre and Cleveland, 2012). A centrosome consists of two components: a pair of centrioles and the surrounding pericentriolar matrix (PCM). After replication of the centrioles, the longer centriole (mother centriole) engages with the shorter one (daughter procentriole) in a V-shape. When a cell enlarges in the G_2_ phase, the short daughter procentriole undergoes elongation to a certain length before the subsequent M phase. A single centriole consists of a microtubule doublet or triplet, which is equivalent to the cytoplasmic microtubule. Several factors localised on centrioles have been shown to be involved in the centriole elongation process (Delgehyr et al., 2012; Mottier-Pavie and Megraw, 2009; Saurya et al., 2016; Schmidt et al., 2009). The most critical step in centrosome duplication is the duplication of centrioles, which requires stringent regulation. However, the entire mechanism underlying regulation of centriole elongation and the regulatory factors required for the process are not known. Among the centriole-associated proteins, those belonging to the kinesin-13 family are known to act as microtubule-severing kinesins (Ems-McClung and Walczak, 2010). Klp10A, a *Drosophila* member of the family, has been shown to play an indispensable role in the regulation of centriole elongation (Delgehyr et al., 2012). Based on these observations, we speculated that some of the factors regulating microtubule length might overlap with those required for centriole elongation. It is possible that the production of centrioles of specific length requires a balance between polymerisation and depolymerisation of the triplet microtubules. However, the main factor(s) counteracting Klp10A and promoting centriole elongation remain to be identified.

Another characteristic complex containing Cp110 is localised at the distal tip of the centriole, where it regulates the accessibility of the distal end to the shrinking and hypothetical lengthening factors (Chen et al., 2002; Nigg and Raff, 2009), thereby regulating centriole elongation at this end (Chen et al., 2002; Schmidt et al., 2009; Spektor et al., 2007). In the absence of Klp10A, the longer centrioles harbour incomplete ninefold symmetry at their ends in *Drosophila* cultured cells and tend to undergo fragmentation (Delgehyr et al., 2012). Importantly, *Cp110* depletion differentially affects centriole elongation in a species- and/or cell type-specific manner. In *Drosophila* S2 cultured cells, *Cp110* depletion results in centriole length diminution. This effect is rescued by simultaneous depletion of *Klp10A* (Delgehyr et al., 2012). In contrast, *CP110* (also known as *CCP110*) depletion results in centriole elongation in mammalian cells (Schmidt et al., 2009). The centriolar microtubules were dramatically elongated in somatic cells, such as wing discs and larval brain cells, in the *Drosophila* *Cp110*-null mutant, whereas subtle elongation of the structure was observed in the premeiotic spermatocytes of the mutant (Franz et al., 2013).

The premeiotic spermatocyte in *Drosophila* is a good model for investigating centrosomes and centrioles. *Drosophila* spermatogenesis involves four mitotic and two meiotic cycles for the formation of haploid spermatids (Fig. S1; Fuller, 1993; Inoue et al., 2012; Tanabe et al., 2017; White-Cooper, 2004). In the same spermatocyte cyst, each of the 16 cells undergoes synchronous cell growth, which can be divided into the S1 stage, corresponding to S phase, and five subsequent stages, S2 to S6, before initiation of meiosis I. The centrioles, in particular, can be studied more easily in this cell type (Fuller, 1993; Riparbelli et al., 2012; Persico et al., 2019), since these organelles dramatically elongate until the onset of meiosis (Fig. S1C; Riparbelli et al., 2012) and the centriole cylinder is composed of microtubule triplets (Gottardo et al., 2015). In early spermatocytes that possess a pair of centrioles initially, centrioles duplicate at S1 stage. As primary spermatocytes enter in the growth phase, centrioles migrate toward the surface where they assemble the primary cilium at the distal end of basal body (Fig. S1B). At the beginning of meiotic division I, centrioles move close to the nucleus with their associated ‘membrane pocket’ on the distal end of the cilium-like region (CLR; Fig. S1C). Between the CLR and the basal body there is the transition zone (TZ), which plays an important role in elongating the primary cilium of the spermatocyte (Vieillard et al., 2016). Centrioles are no longer duplicated between the two meiotic divisions. Primary spermatocytes hold two pairs of centrioles composed of nine triplet microtubules and engaged by a cartwheel structure at the proximal ends (Fig. S1C). The centriole pair is disengaged during prophase II, and, consequently, singlet centrioles organise the centrosomes of secondary spermatocytes.

Previous studies have shown that Orbit (the *Drosophila* CLASP orthologue, encoded by *chb*) is essential for microtubule polymerisation, as it adds tubulin dimers to the plus end of the microtubules (Inoue et al., 2000, 2004; Lemos et al., 2000; Maiato et al., 2005); however, its role in centriole elongation has not been examined. Hence, in this study, we aimed to investigate whether Orbit was involved in centriole elongation in the mature premeiotic spermatocytes before male meiosis. As Orbit antagonises Klp10A, a severing factor determining the length of spindle microtubules in cultured *Drosophila* cells (Laycock et al., 2006), we assessed whether Orbit was also involved in centriole length regulation.

In addition, we highlighted the importance of these regulators of centriole dynamics and the distal end capping proteins in the centriole elongation process using *Drosophila* spermatocytes. We also discuss the importance of regulating the elongation of duplicated centrioles to a certain length for proper chromosome inheritance during male meiotic divisions.

## RESULTS

### Differential distribution of several centriole-associated proteins along the centrioles in *Drosophila* premeiotic spermatocytes

**Fig. 1.**
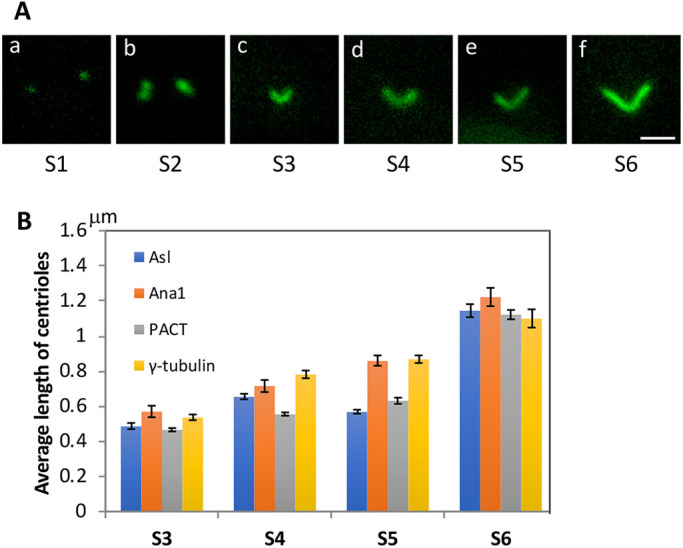
**Centriole elongation as a progression of spermatocyte growth during the growth stages of premeiotic spermatocytes before male meiosis.** (A) Fluorescence observation of a pair of centrioles in premeiotic spermatocytes expressing Asl–YFP, imaged using a confocal microscope. The centrioles in the spermatocytes at stages S1 to S6 (prophase I) during the growth phase were observed. Scale bar: 1 µm. (B) Average length of the centriole structures visualised by immunostaining with anti-Asl or anti-γ-tubulin antibodies, or by expression of Ana1–GFP or mRFP–PACT, during the later stages of the growth phase (S3–S6), in which centrioles elongate distinctively. Data are mean±s.e.m. from *n*=48 cells.

To understand the mechanism by which centrioles of specific lengths are generated, we used *Drosophila* primary spermatocytes to observe centrioles before meiosis, and to identify factors involved in the elongation processes. Initially, we performed immunostaining of premeiotic spermatocytes using an antibody against centriolar protein Asl (Asterless), to observe centrioles from the S1 phase to prophase I during the growth stage before male meiosis. Conventional fluorescence microscopy revealed that a pair of centrioles gradually elongated to attain the length of the mature centrioles during the growth stage (Fig. 1A). Hence, we next visualised the centrioles using four markers, Asl, Ana1, γ-tubulin, and PACT (pericentrin-AKAP450 centrosomal targeting domain, the C-terminal domain of the *Drosophila* PCM protein Plp), which are centriole-associated proteins. We measured the average length of the organelles from the S3 stage, when prominent cell growth has been initiated, to the initiation of meiosis I (Fig. 1B). In each case, the centrioles observed using these markers elongated to ∼1.1 µm on average before and/or at the beginning of meiosis I. This is twice as long as the length observed at the S3 stage. This encouraged us to investigate the mechanism of centriole elongation in premeiotic spermatocytes. To confirm centriole elongation in spermatocytes, we observed the cells using structured illumination microscopy (SIM). First, we observed the distribution of four known centriole-associated proteins – Asl, PACT, β-tubulin and γ-tubulin – on two pairs of centrioles in the premeiotic spermatocyte in the mature stage by expressing the fluorescently tagged proteins or immunostaining with respective antibodies. We observed spermatocytes expressing GFP–β-tubulin and mRFP–PACT (Fig. S2A). The mRFP–PACT-expressing cells were also investigated through immunostaining for γ-tubulin (Fig. S2B) or Asl (Fig. S2C). These four proteins localised on the centrioles, although their distributions differed (Fig. S2A–C″). Both Asl and γ-tubulin were distributed on the basal body composed of triplet microtubules in both centrioles of a pair (Fig. S2B,C). Asl was distributed along the whole basal body region, whereas the anti-γ-tubulin signal appeared on the basal body as a dotted pattern (Fig. S2B,B′,C,C′). These results were consistent with those of previous reports (Lattao et al., 2017; Moritz et al., 1995). However, these two proteins are not suitable for specifically measuring centriole length, as they localise both on centrioles and in the PCM region. The β-tubulin distribution represents doublet and triplet microtubules in the centrioles (Fig. S2A,A′). The average length of the β-tubulin region on the centrioles was 1.08±0.03 µm (mean±s.e.m.; *n*=20). Although the tubulin was distributed along the entire centriole, it also constitutes the subcellular microtubules, which renders specific observation of centrioles difficult. In contrast, PACT is known to localise along the centrioles (Gillingham and Munro, 2000; Jana et al., 2018; Richens et al., 2015). PACT distribution almost overlapped with the β-tubulin region on centrioles, although it was shifted slightly toward the proximal end of the centrioles compared to the β-tubulin region (>50 premeiotic spermatocytes from 10 males were examined; Fig. S2A–A″). The PACT region was 1.10±0.02 µm in length (mean±s.e.m.; *n*=54), which was as long as that of the β-tubulin region. Therefore, we used mRFP–PACT as a marker to visualise the basal body of centrioles and measure their length.

### Orbit, a microtubule polymerisation factor, localises along the centriole basal body and predominantly accumulates at the distal tip of centrioles

**Fig. 2.**
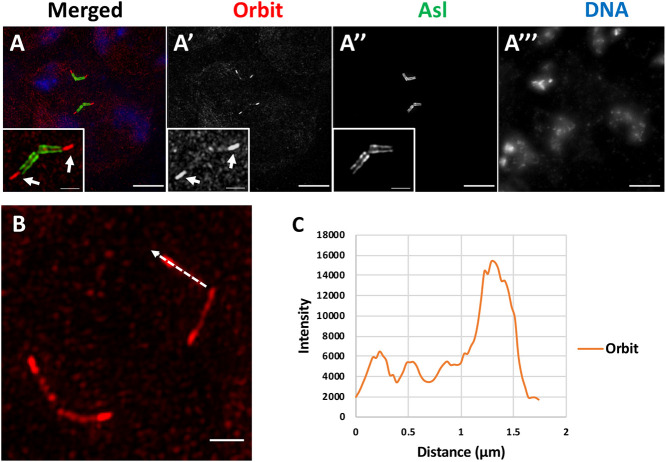
**Centriolar localisation of Orbit protein in premeiotic spermatocytes.** (A,B) Observation of premeiotic spermatocytes immunostained with anti-Orbit antibody and imaged using SIM. (A) Two pairs of centrioles in a premeiotic spermatocyte. Images show anti-Orbit immunostaining of the spermatocyte (red in A, white in A′), anti-Asl immunostaining (green in A, white in A″) and DNA staining by DAPI (blue in A, white in A‴). Insets show a pair of the engaged centrioles. Arrows indicate the distal ends of centrioles. Fluorescence images show the typical distribution of proteins and are representative of 60 centrioles imaged in 15 premeiotic spermatocytes. (B) A magnified view of a pair of centrioles stained with anti-Orbit antibody in the wild-type spermatocyte. Dashed arrow indicates the centriolar region used for line analysis in C. (C) Line analysis of the anti-Orbit immunofluorescence along one of the pair of centrioles from the proximal end (distance=0 μm) to the distal end. The graph indicates enrichment of Orbit around the distal tip of the centriole. Scale bars: 5 μm in A–A‴, 1 μm in A–A″ insets, 1 μm in B.

Orbit is well known as an essential factor for microtubule polymerisation that acts by adding α- and β-tubulin heterodimers at the plus end of the microtubules. To determine whether Orbit is also involved in elongation of the triplet microtubules, we first performed simultaneous immunostaining of wild-type spermatocytes with anti-Orbit and anti-Asl antibodies. Asl was localised on the basal body structure composed of triplet microtubules in the paired centrioles (Fig. 2A,A″; Fig. S2C′). In contrast, Orbit was observed along the centrioles, while the inside of the basal body structure was visualised using anti-Asl immunostaining (Fig. 2A,A′). Importantly, Orbit was especially enriched at the distal end of centrioles, projecting from the Asl-localisation region (arrows in Fig. 2A,A′). Line analysis quantitating the fluorescence confirmed the protein distribution (Fig. 2B,C). Next, the predominant localisation at the distal end encouraged us to investigate whether Orbit accumulates at the TZ of the centrioles (Fig. S1B, Fig. S3). We induced expression of GFP–Orbit in the cells expressing a TZ protein with a fluorescent tag, Cby–Tomato. Cby was localised only at the most distal part of the Orbit-localising region (Fig. S3C–C″). These observations suggest that Orbit overexpression does not result in excess elongation of the TZ region. These localisation data, together with the known microtubule polymerisation role of Orbit, suggest that Orbit is possibly involved in elongation of the centriole at the distal end in spermatocytes before meiosis.

### Orbit is essential for centriole elongation in the spermatocytes before male meiosis

**Fig. 3.**
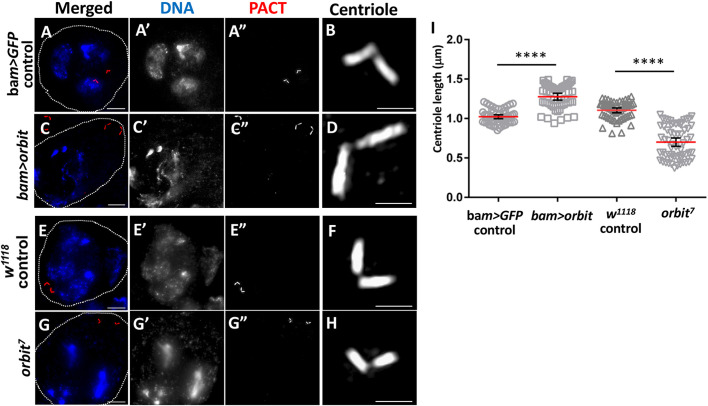
**Orbit plays an essential role in elongating centrioles to a certain length in premeiotic centrioles.** (A–H) Observation of centrioles in premeiotic spermatocytes either harbouring spermatocyte-specific overexpression of Orbit or homozygous for the *orbit^7^* hypomorphic mutation, imaged using SIM. Centrioles were visualised using mRFP–PACT expression (red), DNA was stained with DAPI (blue). (A–A″) Centrioles in a control spermatocyte (*bam>GFP*). (C–C″) Centrioles in a premeiotic spermatocyte overexpressing Orbit (*bam>orbit*). (B,D) Magnified views of a pair of centrioles shown in A and C, respectively. (E–E″) Centrioles in a control spermatocyte (*w^1118^*). (G–G″) Centrioles in an *orbit^7^* premeiotic spermatocyte. (F,H) Magnified views of a pair of centrioles shown in E and G, respectively. Outer cell boundary of each cell is encircled by a white dotted line. Scale bars: 5 μm in A,C,E,G; 1 μm in B,D,F,H. (I) The length of centrioles in premeiotic spermatocytes overexpressing Orbit and in homozygous cells that have the *orbit^7^* hypomorphic allele, compared with that in control spermatocytes. Data are presented as mean±95% c.i. *n*=53 (*bam>GFP* control), 51 (*bam>orbit*), 54 (*w^1118^* control) and 62 (*orbit^7^*). ****P<0.0001 (Mann–Whitney test).

To investigate this hypothesis, we performed spermatocyte-specific overexpression of Orbit using the *bam-Gal4* driver (*bam>orbit*) and measured centriole length. We defined the PACT-localising region in the centrosome as the centriole region and measured the length in premeiotic spermatocytes at the end of the growth phase before meiosis. We selected premeiotic spermatocytes in the mature stage based on the organisation of DAPI-stained chromatin and cell size. The average length (1.28 μm on average, *n*=51 centrioles) of the centrioles in *bam>orbit* spermatocytes was significantly longer (25%) than that in normal control premeiotic spermatocytes (*bam>GFP*; 1.02 µm, *n*=53) in the mature stage (Fig. 3A–D,I). Moreover, we induced overexpression of Orbit at three different levels using the Gal4/UAS system and compared the average centriole length (Fig. S4A,B). By quantifying total GFP–Orbit fluorescence in each spermatocyte, we confirmed that spermatocytes from males raised at 25°C and harbouring a single copy of *UAS-GFP–Orbit* with *bam-Gal4* had a relatively low level of Orbit overexpression, whereas spermatocytes from males raised at 25°C and carrying *bam-Gal4* and two copies of *UAS-GFP–Orbit* had moderate Orbit overexpression, and those from males raised at 28°C and carrying *bam-Gal4* and two copies of *UAS-GFP–Orbit* had a relatively higher level of Orbit overexpression (Fig. S4C). The length of the centrioles labelled by GFP–Orbit increased with the extent of overexpression. We also confirmed that Orbit overexpression even at a lower level resulted in a significant extension of centrioles labelled by anti-Asl immunostaining (Fig. S4B). Therefore, we conclude that Orbit overexpression can stimulate excessive centriole extension. In the cells overexpressing GFP–Orbit at a moderate level (*w; Cby-Tom/+;bam-Gal4/UAS-GFP–Orbit*), we sometimes observed centriole pairs with longer Orbit-localising regions protruding from basal bodies (Fig. S6C,C′). In these cells, Cby was localised only with the most distal part, not the whole Orbit region. These observations suggest that Orbit overexpression did not result in excessive elongation of the TZ region. Moreover, we also observed the axonemal microtubules extending from basal bodies using anti-acetylated tubulin immunostaining (Fig. S5G), and observed that overexpression of Orbit resulted in excessive elongation of axonemal microtubules extending from the basal bodies.

To verify the involvement of Orbit in regulation of centriole elongation, we measured the length of centrioles in premeiotic spermatocytes homozygous for the hypomorphic allele, *orbit^7^*. Spermatocytes could not be observed in *orbit* mutants homozygous for the null allele due to their death at an earlier stage. The average length of the centrioles (0.70 µm, *n*=62 centrioles) in the hypomorphic mutant was significantly lesser (63% of the control) than that in the control cells (*w^1118^*; 1.10 μm on average, *n*=54 centrioles; Fig. 3E–I). These genetic results suggest that Orbit plays an essential role in both microtubule polymerisation and centriole elongation in spermatocytes.

### Klp10A acts as a negative regulator of centriole elongation in premeiotic spermatocytes

**Fig. 4.**
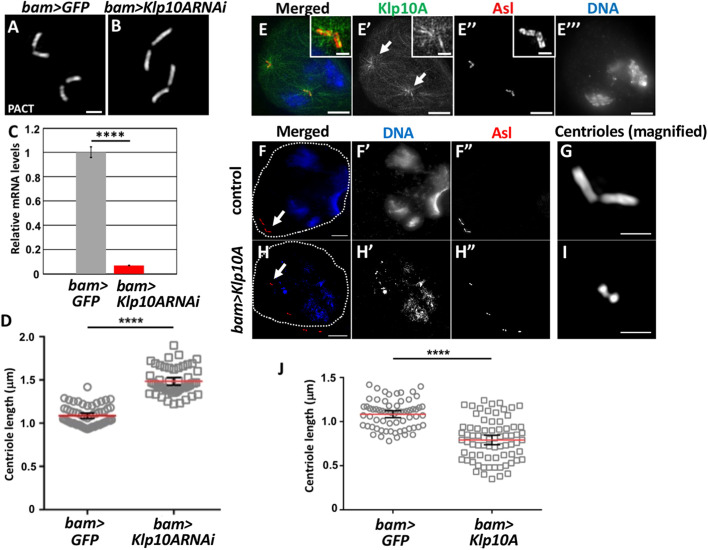
**Centriolar localisation of microtubule-severing factor Klp10A, and the effects of overexpression and depletion of it on centriole elongation in premeiotic spermatocytes.** (A,B) Observation of two pairs of centrioles in premeiotic spermatocytes by mRFP–PACT expression imaged using SIM. (A) Control spermatocyte (*bam>dcr2, GFP*). (B) Spermatocyte depleted of *klp10A* (*bam>dcr2, Klp10ARNAi*). Scale bar: 1 μm. (C) Relative mRNA levels of *Klp10A* in testes expressing *Klp10A* dsRNA (*bam>dcr2, Klp10ARNAi*), assayed using RT-qPCR analysis. *n*=3. (D) Average length of centrioles in control premeiotic spermatocytes (*bam>GFP*) and in premeiotic spermatocytes with spermatocyte-specific depletion of *Klp10A* (*bam>Klp10ARNAi*). A total of 58 centrioles from 18 control premeiotic spermatocytes at mature stage and 52 centrioles from 15 *bam>Klp10ARNAi* cells of the same stage were examined. (E–I) Observation of two pairs of centrioles by SIM. (E–E‴) Centriolar localisation of GFP-tagged Klp10A in a spermatocyte (*bam>Klp10A*; green in E, white in E′) immunostained with the anti-Asl antibody (red in E, white in E″). DNA staining by DAPI (blue in E, white in E‴). Arrows indicate the distribution of Klp10A on the centrioles. Insets indicate a magnified view of one of the two pairs of centrioles in the cell. Scale bars: 5 μm, 1 μm insets. (F–F″,H–H″) Two paired centrioles in premeiotic spermatocytes, visualised by anti-Asl immunostaining (red in F,H; white in F″,H″). DNA staining by DAPI (blue in F,H; white in F′,H′). (F–F″) Control (*bam>GFP*) spermatocyte. (H–H″) Spermatocyte overexpressing Klp10A (*bam>Klp10A*). Arrows indicate centriole pairs shown in G and I. Outer cell boundary of each cell is encircled by a white dotted line. Scale bars: 5 μm. (G,I) Magnified images of a pair of centrioles shown in F and H, respectively. Scale bars: 1 μm. (J) Average length of centrioles in premeiotic spermatocytes. *n*=74 (*bam>Klp10A*) and 67 (*bam>GFP*). In C,D,J, data are presented as mean±95% c.i. ****P<0.0001 (Mann–Whitney test).

A previous study has shown that mutation in *Klp10A* leads to production of longer centrioles in *Drosophila* primary spermatocytes (Delgehyr et al., 2012). We confirmed that Klp10A localised on centrioles in a premeiotic spermatocyte (Fig. 4E–E″, also see insets in the panels). Next, we induced spermatocyte-specific depletion of *Klp10A* using dsRNA against its mRNA, together with expression of *dcr2* to raise the RNAi efficiency. Results of RT-qPCR confirmed that *UAS-Klp10ARNAi* efficiently depleted the *Klp10A* mRNA [<10% of the control (*bam>dcr2, GFP*) level; Fig. 4C]. In *Klp10A*-depleted spermatocytes (*bam>dcr2, Klp10ARNAi*), the average length of centrioles (1.48 μm, *n*=52 centrioles; Fig. 4C,D) was 137% of the length of centrioles in control spermatocytes (1.09 μm, *n*=58 centrioles; Fig. 4A,B). *Klp10A* depletion in the spermatocytes resulted in the production of significantly longer centrioles. Conversely, the average length of centrioles in premeiotic spermatocytes overexpressing Klp10A (*bam>Klp10A*) was 0.79 μm (*n*=74 centrioles), which was 73% of the average length (1.08 μm, *n*=67 centrioles) in normal spermatocytes (*bam>GFP*) (Fig. 4F–J). These results were consistent with the previous finding that Klp10A acts a negative regulator of centriole length in premeiotic spermatocytes (Delgehyr et al., 2012).

### Simultaneous overexpression of Orbit and depletion of *Klp10A* results in additive enhancement of centriole elongation

**Fig. 5.**
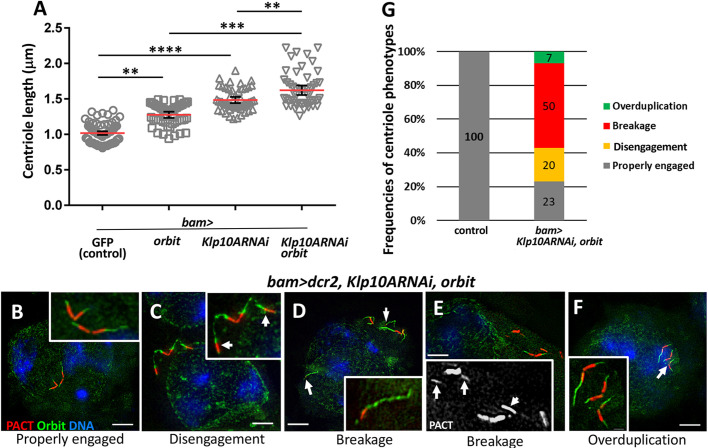
**Overexpression of Orbit in spermatocytes depleted of *Klp10A* has an additive effect on centriole elongation and results in a loss of integrity of the centrioles.** (A) Average length of centrioles in the premeiotic spermatocytes having overexpression of Orbit and depletion of *Klp10A* (*bam>dcr2, Klp10ARNAi*, *orbit*). The centrioles of the cells with each of the indicated genotypes were visualised by expression of mRFP–PACT and their length was measured. Data are presented as mean±95% c.i. More than 50 centrioles from 14 cells among the premeiotic spermatocytes at mature stage were examined. First column, control; second column, spermatocytes overexpressing Orbit; third column, depletion of *Klp10A*; fourth column, overexpression of Orbit and depletion of *Klp10A*. Note that there is an additive effect on the centriole elongation. Significance was tested by Kruskal–Wallis test and Mann–Whitney test. **P<0.01, ***P<0.001, ****P<0.0001 (Mann–Whitney test). (B–F) Observation of centrioles in spermatocytes having *orbit* overexpression and *Klp10A* depletion (*bam>dcr2, Klp10ARNAi, orbit*) by SIM. GFP–Orbit (green), PACT (red) and DNA (blue) are shown. (B) Spermatocyte having the centrioles engaged properly, with an overly longer cilium-like structure labelled by GFP–Orbit. Inset shows a magnified image of the centrioles. (C–F) Several types of abnormal centriole structure seen in the premeiotic spermatocytes. Arrows indicate each example of abnormal centrioles. Insets in C–F indicate magnified images of the abnormal centriole pairs. (C) Centrioles disengaged. (D,E) Centrioles that have lost centriolar integrity (breakage). Arrows indicate smaller pieces of basal body, associated with elongated cilia labeled by GFP–Orbit. Inset in D shows a magnified image of the abnormal centriole indicated by the left-hand arrow. Inset in E shows PACT-positive pieces indicating a thinner basal body-like structure. (F) Overduplication of centrioles. Scale bars: 5 μm. (G) Frequencies of spermatocytes having properly engaged centrioles and abnormal centrioles of each category. Control: *bam>dcr2, LacZ*. Grey, properly engaged; yellow, disengagement; red, breakage; green, overduplication. Percentages in each category are indicated. A total of 11 control spermatocytes and 30 *bam>dcr2, Klp10ARNAi*, *orbit* spermatocytes were analysed.

Next, we investigated whether Orbit and Klp10A interacted and antagonised each other. Toward this purpose, we overexpressed Orbit and depleted Klp10A simultaneously in spermatocytes (*bam>dcr2, Klp10ARNAi, orbit*) and measured the centriole length (Fig. 5A). Interestingly, spermatocytes harbouring Orbit overexpression and Klp10A depletion possessed an overly elongated GFP–Orbit signal protruding from the distal end of the basal body. Orbit was distributed along centrioles overly extended from the basal body and predominantly accumulated at the distal end of the centrioles (Fig. 2B, Fig. 5B). In spermatocytes, the effects of the modified expression of these two genes on centriole elongation were additive (1.62 μm average length, *n*=54 centrioles) compared to those in *bam>orbit* (1.27 μm average length, *n*=51 centrioles) and *bam>dcr2, Klp10ARNAi* (1.48 μm average length, *n*=52 centrioles) (Fig. 5A). These results suggest that these two factors act antagonistically for the production of centrioles of specific length.

### Loss of centriole integrity is frequently observed in premeiotic spermatocytes with both Orbit overexpression and *Klp10A* depletion

In addition to elongation of centrioles in spermatocytes with both Orbit overexpression and *Klp10A* depletion, we observed many centrioles with abnormal structure in these spermatocytes. Control spermatocytes (*bam>dcr2,LacZ*) contained a pair of properly engaged centrioles (100%, *n*=11). Surprisingly, in spermatocytes harbouring both *Klp10A* depletion and Orbit overexpression (*bam>dcr2, Klp10ARNAi, orbit*), loss of centriole integrity was frequently observed in 77% of cells (Fig. 5G). We classified the abnormal centrioles into three classes: disengagement, breakage and overduplication. When we observed an abnormal cell with two or four unpaired centrioles of full length (∼1 µm or longer), we considered that disengagement of either or both pairs of centrioles occurred precociously before meiosis. Thus, we categorised the cell as having a disengagement phenotype. If we found a cell with unpaired centriole pieces shorter than 1 µm, we classified it as having a breakage phenotype, rather than disengagement. Cells carrying extra pairs or single full-length centrioles were classified as having an overduplication phenotype. Shorter centrioles in *orbit^7^*-mutant cells and *bam>Klp10A* cells were not included in the loss-of-integrity phenotypes, as far as they were engaged. In addition, we observed spermatocytes showing two different phenotypes at the same time. For example, we found cells showing both disengagement and breakage phenotypes (Fig. 5E). To avoid counting these cells twice, we categorised them as having the breakage phenotype. When we categorised cells showing both breakage and overduplication phenotypes, we assigned these to the overduplication category. Cells showing disengagement and overduplication phenotypes were assigned to the overduplication category. In total, 20% of *bam>dcr2, Klp10ARNAi, orbit* spermatocytes contained abnormal sets of centrioles that were precociously separated (disengagement, Fig. 5C). Half of the cells at mature stage harboured abnormally shorter pieces of the centrioles (breakage, Fig. 5D). In addition, 7% of cells contained excessively duplicated centrioles (overduplication, Fig. 5F). Only 23% of the cells possessed properly engaged centrioles (Fig. 5B). These findings suggest that excessively elongated centrioles may easily lose their integrity.

### A reduction or loss of *Cp110* results in production of slightly longer centrioles and enhances the centriole elongation phenotype caused by Orbit overexpression

Cp110 is a member of the distal tip complex involved in regulation of centriole elongation. First, we confirmed that Cp110 was localised on centrioles at the distal end in the earlier stages of premeiotic spermatocytes, but not at later stages (Fig. S6A–D). Orbit colocalised with Cp110 on distal tips of the centriole basal body at early stage (Fig. S6A). Until mid-stage, Cp110 was localised to the most distal area of the Orbit-localising region (Fig. S6B). The GFP–Orbit signal protruding from the distal end of centrioles overlapped with the anti-acetylated tubulin immunostaining signal (Fig. S5G). Therefore, GFP–Orbit was possibly distributed to axonemal microtubules emanating from the distal tips of the basal body (Fig. S5B–D). Cp110 disappeared from centrioles at late spermatocyte stage, whereas Orbit continued to be localised on centrioles (Fig. S6C,D). A previous study reported that Cp110 colocalises with Klp10A at the distal ends in *Drosophila* cultured cells (Delgehyr et al., 2012). We performed a proximity ligation assay (PLA) and showed that Orbit and Cp110 are closely associated with each other at the end of the centrioles in earlier, but not later stage spermatocytes (Fig. S7).

Thus, we next investigated whether these proteins are interdependent on each other in centriole localisation. We first performed anti-Cp110 immunostaining of control (Fig. S8A–C) and *orbit^7^* mutant spermatocytes (Fig. S8D–F) during spermatocyte development. Cp110 was localised at the distal ends of centrioles in control spermatocytes at the early and mid-stages [89.3% (75/84) of the cells, no signals in 10.7% (9/84)]. In the hypomorphic mutant cells at the same stages, the protein was comparably observed in 82.7% (91/110) of the centrioles [no signals in 17.3% (19/110)]. Conversely, we investigated whether Orbit is localised at the distal centriole ends in spermatocytes from *Cp110*-null mutant males. Among 66 centrioles in control spermatocytes expressing GFP–Orbit, the signal was present at distal ends of 97.0% (64/66) of centrioles [no signal in 3% (2/66)]. Similarly, among 85 centrioles in spermatocytes at mid- and late stages from the *Cp110*-null mutant with GFP–Orbit expression, the signal was still localised at distal ends on 98.8% (84/85) of the centrioles [1.1% (1/85) possessed no signal at either end of two pairs; Fig. S8G]. Therefore, we speculate that Orbit and Cp110 are not mutually dependent for their localisation on centrioles.

**Fig. 6.**
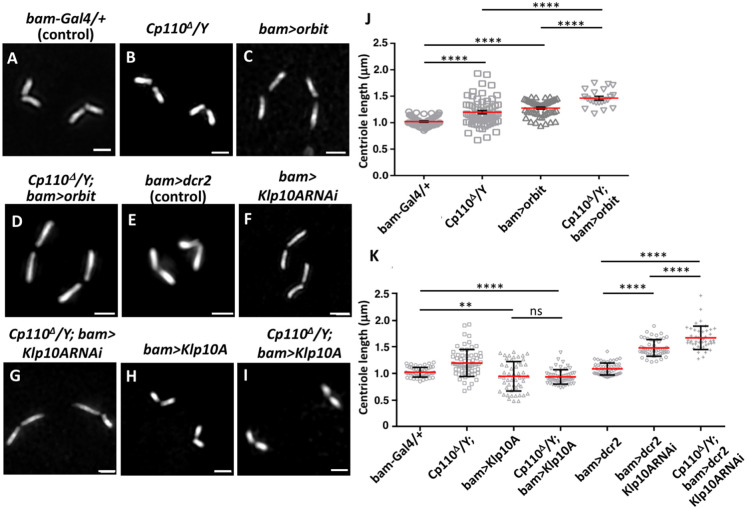
**Effects of *Cp110*-null mutation on centriole elongation in spermatocytes overexpressing either Orbit or Klp10A, or in** **those harbouring Klp10A depletion.** (A–I) Observation of centrioles labelled by mRFP–PACT using SIM. Two sets of centrioles in (A) a control spermatocyte (*bam-Gal4/+*), (B) a *Cp110*-null mutant spermatocyte (*Cp110^Δ1^/Y*), (C) a spermatocyte overexpressing *orbit* (*bam>orbit*), (D) a *Cp110*-null mutant spermatocyte overexpressing *orbit* (*Cp110^Δ1^/Y*; *bam>orbit*), (E) a control spermatocyte (*bam>dcr2*), (F) a spermatocyte depleted of *Klp10A* (*bam>dcr2, Klp10ARNAi*), (G) a *Cp110*-null mutant spermatocyte depleted of *Klp10A* (*Cp110^Δ1^/Y*; *bam>Klp10ARNAi*), (H) a spermatocyte overexpressing *Klp10A* (*bam>Klp10A*), and (I) a *Cp110*-null mutant spermatocyte overexpressing *Klp10A* (*Cp110^Δ1^/Y*; *bam>Klp10A*). Scale bars: 1 µm. (J) Average length of centrioles in premeiotic spermatocytes at mature stage for the four indicated genotypes. (K) Average length of centrioles in premeiotic spermatocytes at mature stage for the seven indicated genotypes. Data in J,K are presented as mean±95% c.i. *n*>21 (J) and *n*>45 (K). **P<0.01; ****P<0.0001; ns, not significant (two-tailed unpaired Student's *t*-test).

Next, we investigated whether the distal end complex protein is required for centriole elongation, using the *Cp110-*null mutant, *Cp110^Δ1^*. A previous study has reported that most centrioles of *Cp110^Δ1^* mutant spermatocytes and embryos behave normally (Franz et al., 2013). By contrast, we showed that spermatocytes from the *Cp110^Δ1^* mutant possessed slightly longer centrioles (1.20 µm on average, *n*=66 centrioles in 19 spermatocytes) than the control (1.10 µm; Fig. 6A,B,J). Among 66 centrioles, the longest centriole was 1.93 µm in length and the shortest was 0.67 µm. In total, nine centrioles were longer than 1.5 µm (Fig. 6J).

If the distal tip protein acts as a cap that restricts the access of factors dynamically regulating centriole length, we would expect the loss of *Cp110* to enhance the phenotype of overly longer centrioles in cells overexpressing Orbit. In the *Cp110*-null mutant, the average length of centrioles increased (1.20 µm on average, *n*=66 centrioles examined) compared to that in control spermatocytes (1.02 µm, *n*=53). Compared to the length of centrioles in cells overexpressing Orbit (*bam>orbit*; 1.33 µm, *n*=49) or in the *Cp110^Δ1^* mutant cells, the centriole length was significantly increased in *Cp110^Δ1^/Y; bam>orbit* cells (Fig. 6A–D,J; 1.46 µm, *n*=21; *P*<0.0001 in both cases, Student's *t*-test). Consistently, the centrioles in *Cp110^Δ1^/Y; bam>Klp10ARNAi* cells were also significantly increased in length (1.67 µm, *n*=45; Fig. 6G–I,K) compared to those in cells harbouring Klp10A depletion (*bam>Klp10ARNAi*; 1.48 µm, *n*=52, *P*<0.0001 in both cases; Fig. 6E–G,K). Contrary to the conditions that stimulate centriole elongation, the centriole length did not change in *Cp110^Δ1^* cells overexpressing Klp10A (*Cp110^Δ1^/Y; bam>Klp10A*; 0.94 µm, *n*=55) compared to that in *bam>Klp10A* cells (0.95 µm, *n*=51; Fig. 6H,I,K), even though centrioles decreased in length in both cases (Fig. 6K). These results suggested that *Cp110* is also involved in determination of centriole length by restricting the access of these dynamic factors at the distal ends.

### The *Cp110*-null mutation substantially enhances the loss of centriole integrity phenotype in spermatocytes harbouring Orbit overexpression or *Klp10A* depletion

**Fig. 7.**
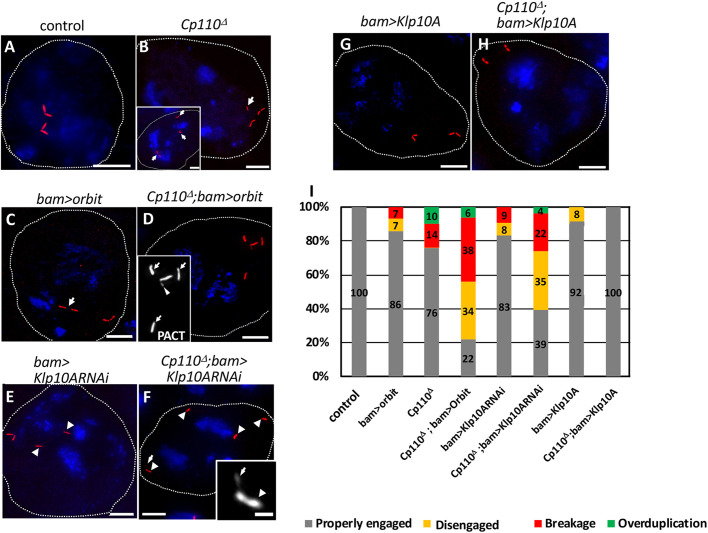
**Abnormal centriole structures observed in premeiotic spermatocytes of the *Cp110*-null mutant and mutant cells overexpressing Orbit or having *Klp10A* depletion.** (A–H) Observation of premeiotic spermatocytes at mature stage carrying two pairs of centrioles labelled by mRFP–PACT (red), imaged using SIM. DNA was stained with DAPI (blue). (A) Control spermatocyte (*w^1118^*) containing two pairs of engaged centrioles. (B) Spermatocyte hemizygous for a *Cp110*-null mutation, *Cp110^Δ1^*, containing a centriole piece (arrow) with two normal-looking pairs of centrioles. Inset shows three pairs of engaged centrioles (arrows) in another spermatocyte of the same genotype. (C) Spermatocyte overexpressing Orbit (*bam>orbit*) containing disengaged centrioles (arrow) and a pair of engaged centrioles. (D) *Cp110^Δ1^* spermatocyte with Orbit overexpression (*Cp110^Δ1^; bam>orbit*). Inset shows a magnified view of unpaired centrioles (arrows) and a small centriole piece (arrowhead). (E) Spermatocyte with *Klp10A* depletion (*bam>dcr2, Klp10ARNAi*) with unpaired centrioles (arrowheads). (F) *Cp110^Δ1^* spermatocyte with *Klp10A* depletion (*Cp110^Δ1^; bam>dcr2, Klp10ARNAi*) with unpaired centrioles (arrowheads) and a small centriole piece (arrow). Inset shows a magnified view of an unpaired centriole (arrowhead) and the small piece (arrow). (G,H) Spermatocytes containing normal two pairs of engaged centrioles. (G) Spermatocyte overexpressing Klp10A (*bam>Klp10A*). (H) *Cp110^Δ1^* spermatocyte overexpressing Klp10A (*Cp110^Δ1^; bam>Klp10A*). Outer cell boundary of each cell is encircled by a white dotted line. Scale bars: 5 μm. (I) Frequencies of spermatocytes of each genotype having properly engaged centrioles and abnormal centrioles. *n*>12 spermatocytes were analysed for each genotype. Grey, properly engaged; yellow, disengaged; red, breakage; green, overduplication.

In addition to moderately excessive elongation of centrioles, we observed loss of centriole integrity in 24% of the *Cp110*-null mutant spermatocytes (*n*=21, Fig. 7I). These centriole phenotypes were similar to those seen in the cells harbouring Orbit overexpression and Klp10A depletion simultaneously. They were classified by comparison with control centrioles (Fig. 7A) into centriole breakage, inferred from a presence of shorter pieces labelled by mRFP–PACT (arrow in Fig. 7B, Fig. 7I; 4%, 3 of 21 cells), and centriole overduplication (arrows in inset of Fig. 7B, Fig. 7I; 10%, 2 of 21 cells). Surprisingly, we observed loss of entriole integrity in *Cp110^Δ1^* cells harbouring Orbit overexpression at significantly higher frequency (78% of the centrioles in the 32 spermatocytes observed), compared to the frequency of loss-of-integrity phenotypes in either *bam>orbit* cells (Fig. 7C) or *Cp110^Δ1^* cells (Fig. 7I). We observed several types of abnormal cells containing the following centriole abnormalities: premature separation of the centriole pair (disengagement; arrows in Fig. 7D, Fig. 7I; 34%), putative centriole fragments (breakage; arrowhead in Fig. 7D, Fig. 7I; 38%), and presence of extra pairs of centrioles (overduplication; Fig. 7I, 6%,). These centriole phenotypes were also observed in the *bam>dcr2, Klp10ARNAi, orbit* spermatocytes (Fig. 5C–F). Consistently, these centriole integrity phenotypes were seen in *Cp110^Δ1^; bam>dcr2, Klp10ARNAi* spermatocytes (*n*=23); 35% of cells harboured prematurely separated centrioles (arrowheads in Fig. 7F), 22% of cells contained shorter centrioles (arrow in Fig. 7F), and 4% of cells had extra pairs of centrioles, while 39% of spermatocytes contained normally engaged centrioles (Fig. 7F,I). By contrast, disengaged centrioles (8%, arrowheads in Fig. 7E) and shorter centrioles (9%) were observed at lower frequencies in *bam>dcr2*, *Klp10ARNAi* spermatocytes (Fig. 7I). We observed the centriole integrity phenotypes in the *Cp110^Δ1^* cells with Klp10A depletion at much higher frequencies (61% of the centrioles), compared to those in either *bam>orbit* cells or *Cp110^Δ1^* cells (Fig. 7I). Conversely, only a few abnormal centrioles were observed in spermatocytes overexpressing Klp10A (*n*=12; Fig. 7G,I), and abnormal centrioles were not observed in *Cp110*-mutant cells overexpressing Klp10A (*n*=15; Fig. 7H,I)*.*

Moreover, we investigated whether posttranslational modifications of tubulins were indeed affected by depletion of the *Cp110* gene or by the null mutation. In the absence of another component of the distal end complex, Cep97, tubulin acetylation is inhibited (Dobbelaere et al., 2020). Acetylated tubulin foci appeared on the distal ends of centrioles in control spermatocytes at mid-stage (Fig. S5A) and became elongated at late stage (Fig. S5B). We did not observe foci at the distal ends in the *Cp110*-null mutant cells (Fig. S5C,D). These observations were consistent with the published results in the *Cep97*-null mutants (Dobbelaere et al., 2020). Furthermore, we examined whether Orbit overexpression or Klp10A depletion influences tubulin acetylation in the absence of Cp110. Interestingly, we found that acetylated microtubules excessively grew from the distal ends in both cases (arrowheads in Fig. S5E–G), indicating that modification and elongation of the stabilised microtubules was not perturbed in altered expression of Orbit or Klp10A without Cp110. Rather, these genetic alterations resulted in enhanced elongation of axonemal microtubules.

### Abnormal centrioles associate with multipolar spindles in meiotic cells derived from *Cp110*-null mutant spermatocytes overexpressing Orbit

**Fig. 8.**
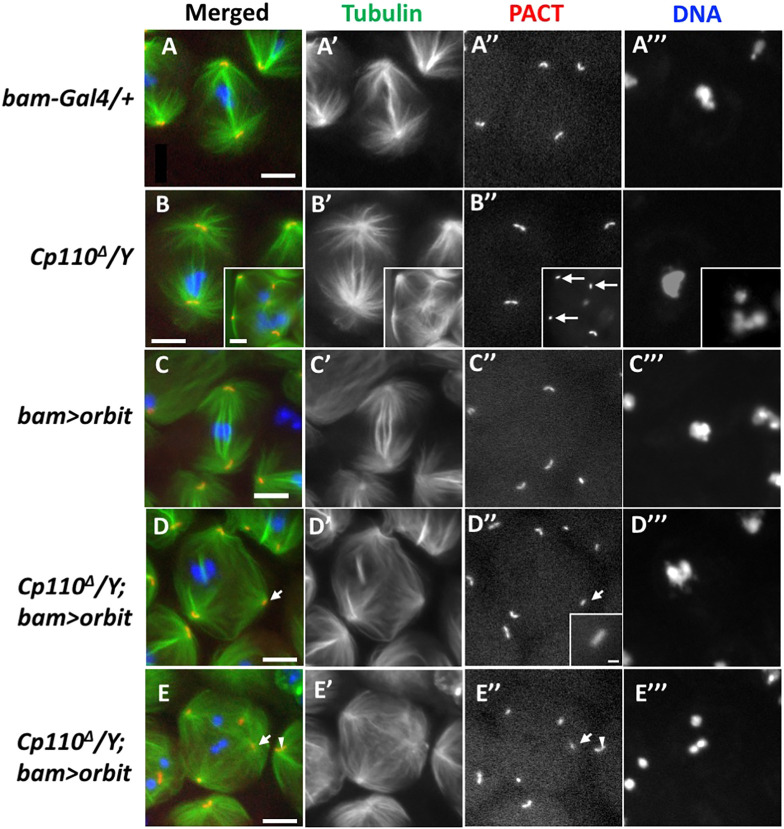
**Multipolar spindle microtubule structures** **in metaphase I spermatocytes of *Cp110*-null mutant males overexpressing Orbit.** (A–E‴) Fluorescence micrographs of spermatocytes at metaphase I, showing (A–A‴) control male spermatocytes (*bam-Gal4/+*), (B–B‴) *Cp110* mutant spermatocytes (*Cp110 ^Δ1^/Y*), (C–C‴) spermatocytes overexpressing Orbit (*w/Y; UAS-GFP–Orbit/+;Ub-mRFP–PACT, bam-Gal4/Ub-β tubulin–GFP*), and (D–E‴) *Cp110^Δ1^* spermatocytes having Orbit overexpression (*Cp110 ^Δ1^/Y; UAS-GFP–Orbit/+;Ub-mRFP–PACT, bam-Gal4/Ub-β tubulin–GFP*). In merged images, microtubules (tubulin–GFP) are shown in green, mRFP–PACT fluorescence is shown in red and DNA is shown in blue (DAPI). Centrioles are visualised in yellow as merged images of tubulin–GFP, mRFP–PACT and (in C,D,E) GFP–Orbit. The primary spermatocytes possess bipolar (A–C‴), tripolar (D–D‴), and tetrapolar (E–E‴) spindle microtubules. Insets in B–B‴ present multipolar spindle microtubules observed at a lower frequency. The meiotic image is associated with centriole fragments (arrows in B″). Inset in panel D″ shows a magnified image of a single unpaired centriole in the spindle pole indicated by an arrow in D,D″. Note that four single centrioles that have already been separated were localised on each pole of the tetrapolar spermatocyte. Arrows in E,E″ show an unpaired centriole, and arrowheads indicate a pair of centrioles in the neighbouring cell. Scale bars: 10 µm.

We next investigated whether the loss of centriolar integrity in the *Cp110*-mutant spermatocytes overexpressing Orbit led to the formation of multipolar spindle microtubules in male meiosis. The control spermatocytes (*w/Y; Ub-mRFP–PACT/+;Ub-GFP–β tubulin/bam-Gal4*) possessed bipolar spindle microtubules in metaphase I (175/175 cells examined from 12 males; Fig. 8A), as did the cells with spermatocyte-specific overexpression of Orbit alone (*w/Y; UAS-GFP–Orbit/+;Ub-mRFP–PACT, bam-Gal4/Ub-GFP–β tubulin*; 153/153 cells examined from 16 males; Fig. 8C). Most of *Cp110*-null mutant spermatocytes (*Cp110^Δ1^*/*Y;Ub-mRFP–PACT, bam-Gal4/Ub-GFP–β tubulin*; 156/159 cells examined from 12 males) showed normal bipolar spindle microtubules in meiosis I (Fig. 8B), although we found a few spermatocytes carrying multipolar spindle microtubules in which pieces of centrioles were associated with each spindle pole at a lower frequency (3/159 cells; inset in Fig. 8B). In contrast, we observed multipolar spindle structures in the *Cp110^Δ1^* mutant cells with spermatocyte-specific overexpression of Orbit at a considerable frequency (10.4%), including 22 cells with tripolar spindles (Fig. 8D) and two cells with tetrapolar spindles among 231 meiosis I cells from 20 males (Fig. 8E). Among 36 spindle poles in 12 tripolar cells, 12 poles harboured paired centrioles, as visualised by mRFP–PACT. By contrast, 24 poles harboured unpaired single centrioles, which corresponded to the disengagement category in Fig. 7I (arrow in Fig. 8D″, magnified view in inset Fig. 8D″). The remaining six multipolar cells possessed two sets of paired centrioles and single shorter centrioles, which corresponded to the breakage category in Fig. 7I (arrow in Fig. 8E). Additionally, we found two tripolar cells, in which every spindle pole (six poles) contained a set of paired centrioles, corresponding to the overduplication category in Fig. 7I. In total, all unpaired centrioles (34/34 examined in 22 multipolar cells) were associated with spindle poles in the abnormal cells. Conversely, we did not observe any free centrioles, which are not associated with spindle poles, in any of 50 bipolar cells or 24 multipolar cells from *Cp110^Δ1^* mutant males overexpressing Orbit. Based on these observations, we conclude that spermatocytes with altered expression of factors regulating centriole dynamics show excess centriole elongation in the absence of the distal end complex. This excess elongation is involved in the production of unpaired and/or fragmented centrioles, subsequently giving rise to abnormal meiotic cells harbouring multipolar spindle microtubules.

## DISCUSSION

### Antagonistic roles of the microtubule polymerising factor Orbit and the depolymerising factor Klp10A in determination of centriole length in spermatocytes

Centrioles in *Drosophila* spermatocytes consist of ninefold triplets of microtubules (Lattao et al., 2017). We showed that the centrioles elongated to a certain length as the cells grew before male meiosis. Klp10A, which is a *Drosophila* kinesin-13 orthologue essential for shortening the microtubules, plays an indispensable role in regulation of centriole length (Delgehyr et al., 2012; this study). Microtubule length can be determined by the balance between polymerisation and depolymerisation of tubulin heterodimers and protofilaments. Thus, dynamic factors that promote microtubule elongation might also play critical roles in the determination of centriole length. In this study, we presented evidence suggesting that overexpression of Orbit results in excessively long centrioles in premeiotic spermatocytes. Conversely, we observed shorter centrioles in hypomorphic *orbit* mutants. These results are consistent with the idea that Orbit is essential for promotion of centriole elongation in spermatocytes. Orbit was initially identified as a microtubule-associated protein (Inoue et al., 2000) that adds tubulin heterodimers at the plus end of microtubules (Maiato et al., 2005; Al-Bassam et al., 2010). Hence, the probability that Orbit might also elongate the triplet microtubules of centrioles by adding tubulin dimers, similar to its function in microtubule elongation, is high. In contrast, the kinesin-13 orthologue Klp10A acts as a microtubule depolymerising factor at the plus ends of microtubules in interphase (Sharp et al., 2005) and is an important regulator of centriole elongation (Delgehyr et al., 2012). In *Drosophila* S2 cells, Klp10A antagonises Orbit in bipolar spindle formation and its maintenance (Laycock et al., 2006). Here, we showed that simultaneous overexpression of Orbit and depletion of *Klp10A* further enhanced centriole elongation. Based on these findings, we proposed that these two factors act antagonistically to produce centrioles of specific length. We observed very long GFP–Orbit signals that extended from basal bodies in spermatocytes overexpressing the protein. Accordingly, we hypothesise that these are overly long axoneme microtubules produced as a consequence of excessively stimulated polymerisation of tubulins by the overexpression. Orbit, like other CLASP proteins, has a microtubule-binding activity to stimulate tubulin polymerisation at the plus end (Inoue et al., 2000; Al-Bassam et al., 2010). Alternatively, we cannot exclude a possibility that Orbit polymerises itself to construct microtubule-like structures on the distal tip of the basal body. It would be interesting to investigate whether basal body and axonemal microtubules overly elongate in cells overexpressing Orbit without a fluorescent tag.

In addition to these two factors regulating centriole microtubules, we hypothesised that Cp110 plays a role as a cap to restrict these factors acting on the distal ends of the microtubules at an earlier stage. After Cp110 releases from the ends at mid-stage, Orbit can access the distal ends more easily and stimulate the centriole microtubules to extend to a certain length. Although the loss of the cap protein resulted in only a subtle extension of centrioles, Orbit overexpression in the absence of Cp110 can change the centriole microtubule dynamics significantly. Consequently, remarkably long centriole microtubules would be produced in the spermatocytes. Consistent with this hypothesis, *Cp110^Δ1^* mutation also significantly enhanced the overly long centriole phenotype in Klp10A-depleted spermatocytes. By contrast, the *Cp110*-null mutation did not enhance the shorter centriole phenotype caused by Klp10A overexpression. Further experiments need to be performed to verify the model. The fact that Cp110 influences centriole length depending on the cell type and cellular context has been demonstrated in previous studies (Delgehyr et al., 2012; Dobbelaere et al., 2020; Franz et al., 2013). It is likely that differential regulation of the conserved core components underlies ciliary basal diversity in different cell types. As argued previously (Jana et al., 2018), cellular-specific and tissue-specific regulation in centriole duplication may be indispensable to regenerate diverse centriole structures.

Previous reports have not investigated whether Orbit and Klp10A are localised on centrioles or around the PCM in *Drosophila* mitotic cells; however, a considerable amount of Orbit accumulates in centrosomes in early embryos, *Drosophila* cultured cells and germline stem cells during *Drosophila* oogenesis and spermatogenesis (Inoue et al., 2000, 2004; Lemos et al., 2000; Máthé et al., 2003). Whether the protein is localised in the PCM or in the centrioles is not clear. Similarly, studies on the cellular localisation of Orbit/CLASP orthologues in other species have also shown centrosome localisation of these proteins. Anti-human CLASP1 immunostaining in Hela cells has demonstrated that the protein is localised on centrosomes during M phase (Maiato et al., 2003). Furthermore, a CLASP orthologue in *Caenorhabditis elegans* has been observed in the centrosomes of its embryonic cells (Espiritu et al., 2012). These reports did not mention whether the orthologues were associated with the centrioles. Recently, it has been reported that Klp10A is dominantly localised in the TZ of the ciliary structures in spermatocytes, spermatids and sensory neurons (Persico et al., 2019). Centrosomal localisation of the protein has also been reported in mitotic cells and germline stem cells in both sexes (Goshima and Vale, 2005; Zou et al., 2008; Radford et al., 2012). Whether Klp10A localises on the cylindrical microtubules of the centrioles or in the PCM of mitotic cells has not been demonstrated. Therefore, whether these antagonistic regulators are localised on centrioles in mitotic cells should be determined at a higher resolution. Furthermore, whether these two regulators are required for centriole length determination in somatic cells warrants further investigation.

### Increased length of centriolar microtubules upon altered expression of the dynamic factors leads to production of disintegrated centrioles and centriole disengagement

In addition to excessively elongated centrioles, we observed several abnormalities in centriole organisation and structure in spermatocytes overexpressing Orbit and/or harbouring *Klp10A* depletion. In the absence of Cp110, the loss-of-centriole-integrity phenotypes were also enhanced. Small pieces of centrioles observed may be broken pieces of over-elongated centrioles, as observed in cancer cells (Marteil et al., 2018). Alternatively, they may have been unpaired centrioles separated precociously from centriole pairs containing daughter procentrioles, which are smaller than normal centrioles (Karki et al., 2017). By contrast, the loss-of-centriole-integrity phenotypes were not observed in cells overexpressing a shortening factor, Klp10A. Hence, we consider that improvised construction of the basal body microtubules may be associated with the loss-of-integrity phenotype; thereby, centriole engagement would be lost. We found centriole fragments with reduced diameter in the centriole microtubules. The presence of disintegrated centrioles supports this idea, but further investigations are necessary to test the current hypothesis.

Spermatocytes homozygous for loss-of-function mutation of *Sas6* and of *Ana2* commonly demonstrate premature centriole separation before meiosis (Stevens et al., 2010; Rodrigues-Martins et al., 2007; Lattao et al., 2017). Hence, Sas6 and Ana2 are considered to be required for centriole engagement and/or maintenance of the pairs. Orbit overexpression and/or Klp10A depletion may affect centriole engagement through interfering with Sas6 (or Ana2) function. It is also possible that the premature disengagement can occur independently of Sas6 or Ana2. In addition, it has been reported that APC/C activation and activation of separate, thereby unexpected, cleavage of Scc1 (also known as RAD21) cohesin can take place in mammalian cultured cells (Karki et al., 2017). We cannot exclude the possibility that alteration of microtubule dynamics in centrioles by altered expression of Orbit and/or Klp10A led to unexpected APC/C activation. This hypothesis will be tested by several experiments in our future work.

### Production of centrioles with constant length is important for centriole integrity and proper meiotic division

Previous studies have also mentioned that *Cp110*-null mutant spermatocytes or syncytial-stage embryos do not show detectable defects in centrosome behaviour, spindle formation or chromosome segregation (Franz et al., 2013). By contrast, we showed in this study that disintegrated centrioles and multipolar spindle microtubules emanating from the centriolar fragments existed in *Cp110*-null mutant spermatocytes and cells overexpressing Orbit, as well as in Klp10A-depleted cells less frequently. Cells homozygous for the loss-of-function *Klp10A* mutation also display multipolar spindle structures (Delgehyr et al., 2012). However, we cannot exclude that the spindle phenotype would result from abnormal microtubule organisation caused by *Klp10A* mutation, rather than centriole disintegration. Surprisingly, in *Cp110* mutants overexpressing Orbit and undergoing meiosis I, we observed that unpaired single centrioles, even a part of them, could act as the MTOC. Centrosomes must be ‘licensed’ to function as an MTOC that nucleates microtubules (Tillery et al., 2018), although the mechanism whereby the ‘license’ is granted remains unclear. Nevertheless, a recent study has reported that excessive elongation of centrioles in cancer cells is related to the generation of overduplicated, fragmented or hyperactive centrosomes that nucleate considerably more microtubules during cell division (Marteil et al., 2018). Chromosome segregation is disturbed in cells harbouring these abnormal centrioles. Interestingly, generation of cells harbouring extra centrosomes has been suggested to be able to drive spontaneous tumorigenesis in mice (Levine et al., 2017; Marteil et al., 2018). Additional studies have reported that excessively elongated centrioles in *Drosophila* spermatocytes affect spermatogenesis via the production of defective flagella (Bettencourt-Dias et al., 2005; Nigg and Raff, 2009). Consistently, we observed immotile sperm production and significant decrease in male fertility in the *Cp110*-mutant males with spermatocyte-specific *Klp10A* depletion (T.S. and Y.H.I., unpublished)*.* These observations suggested that production of excessively elongated centrioles can affect cell division and subsequent spermatogenesis. Once an abnormal spindle microtubule structure is constructed, the extra MTOC results in chromosome mis-segregation and eventually chromosome instability, as observed in cancer cells (Ganem et al., 2009; Marteil et al., 2018).

Certain abnormalities such as mis-segregation of chromosomes and the resulting aneuploidy might appear in the subsequent division of cells containing abnormal centrioles. Conversely, excessively short centrioles may be inadequate as templates for the duplication of centrioles in S phase. Hence, regulation of centrosome length via the antagonistic activities of elongation and shrinking factors, such as Orbit and Klp10A, is important. However, the loss of centriole integrity and the resulting aneuploidy may occur in meiotic cells, but not in mitotic cells, which have stricter microtubule assembly checkpoints. Further investigations are required to understand how centrosome length is regulated in mitotic cells.

Current findings suggest the presence of mutually antagonistic regulation to determine centriole length and the significance of the production of centrioles with a certain length for centriole integrity, and for assurance of proper chromosome segregation. We believe that these findings may enable the identification of a mechanism whereby the loss of centriole integrity causes chromosome mis-segregation in cancer cells.

## MATERIALS AND METHODS

### *Drosophila* stocks

For normal control, *w^1118^* was used. We also used *P*{*UAS-GFP^S65T^*}T2 (DGRC, #106363) and *P*{*UAS-LacZ*} (*Drosophila* Genetic Resource Centre, DGRC; DGRC#107532) as normal controls. To visualise microtubules in spermatocytes, *P*{*Ubi-GFP–β Tubulin*} was used (Inoue et al., 2004). We used the *orbit^7^* hypomorphic allele of the *orbit* gene (Inoue et al., 2004). To overexpress the Orbit protein, *P*{*UASp-GFP–Orbit^#24, #47^*} was used (Miyauchi et al., 2013; Kitazawa et al., 2014). To visualise the centrioles in premeiotic spermatocytes, *P*{*Ub-mRFP–PACT*} (Dix and Raff, 2007), *P*{*Asl–YFP*} (Rebollo et al., 2007), *P*{*GFP–Ana1*} (Blachon et al., 2008), and *P*{*Cby–Tomato*} (Enjolras et al., 2012) were used. These stocks were gifts from Jordan Raff (University of Oxford, Oxford, UK), Cayetano Gonzalez (IRB Barcelona, Barcelona, Spain), Tomer Avidor-Reiss (Harvard Medical School, Boston, MA, USA), and Benedicte Durand (University of Lyon, Lyon, France), respectively. To overexpress Klp10A, *P{EPgy2}EY09320* (Bloomington *Drosophila* Stock Center, BDSC; BDSC#17557) and *P*{*UAS-GFP–Klp10A*} were used. *P{EPgy2}EY09320* carries *P{EPgy2}* inserted 5′ of the *Klp10A* gene in the forward direction of the UAS sequences in the P-element so as to enable induction of Gal4-dependent transcription of the gene (Venken and Bellen, 2007). *P*{*UAS-GFP–Klp10A*} was used to visualise the localisation of GFP-tagged Klp10A in spermatocytes. The stock was kindly distributed by Yukiko Yamashita (University of Michigan, Ann Arbor, MI, USA). For depletion of Klp10A, *UAS-Klp10A RNAi^HMS00920^* (BDSC #33963) was used. These stocks were obtained from BDSC (Bloomington, IN, USA). For depletion of centriolar distal tip factors, Cp110 was used; *UAS-Cp110 RNAi^KK105525^* (#v101161) from Vienna *Drosophila* Resource Center (VDRC; Vienna, Austria) was used. When we induced dsRNA against each mRNA, we also induced co-expression of the *dsr2* mRNA encoding a Dicer2 double-stranded RNA-specific endonuclease in every RNAi experiment to raise the RNAi efficiency in testis cells, where *dcr2* expresses at a lower level (https://flybase.org/reports/FBgn0034246). We used *UAS-dcr2; bam-GAL4::VP16* stock (Hayashi et al., 2016) as spermatocyte-specific Gal4 driver for overexpression various cDNAs and dsRNAs against endogenous mRNAs. We also used *Cp110^Δ1^* as a null allele for the *Cp110* gene (Franz et al., 2013). The *Cp110^Δ1^* stock was kindly distributed by Jordan Raff.

All *Drosophila* stocks were maintained on standard cornmeal food at 25°C, as previously described (Oka et al., 2015). Food: 7.2 g of agar, 100 g glucose, 40 g dried yeast and 40 g of cornmeal was added into 1 litre of water, mixed and boiled while stirring constantly. After the food medium had cooled down, 5 ml of 10% parahydroxybenzonate dissolved in ethanol and 5 ml of propionic acid were added as antiseptics. Gal4-dependent expression was done at 28°C.

### Reverse transcription quantitative PCR analysis

Reverse transcription quantitative PCR (RT-qPCR) analysis was performed to determine mRNA level of various target genes in adult testes. Total RNA was extracted from adult testes using the TRIzol reagent (Invitrogen, Carlsbad, CA, USA). cDNA synthesis from the total RNA was carried out using the PrimerScript II High Fidelity RT-PCR kit (Takara, Shiga, Japan) with random primers. RT-qPCR was performed using FastStart Essential DNA Green Master (Roche, Mannheim, Germany) and a LightCycler Nano (Roche, Basel, Switzerland). We used RP49 as a normalisation reference (Oka et al., 2015). Relative mRNA levels were quantified using LightCycler Nano software version 1.0 (Roche, Basel, Switzerland). The primer sets used were as follows: *Rp49* forward, 5′-TTCCTGGTGCACAACGTG-3′; *Rp49* reverse, 5′-TCTCCTTGCGCTTCTTGG-3′; *Klp10A* forward, 5′-GAATCTAGTCGTCTCGGCCAG-3′; *Klp10A* reverse, 5′-GCTTGTCGGACAGAAGATCGA-3′; *Cp110* forward, 5′-CACGCCTCAACCATTTGTGAA-3′; *Cp110* reverse, 5′-TCGAACTGCAGGATACGATCG-3′. Each sample was duplicated on the PCR plate, and the final results average three biological replicates. For quantification, the ΔΔCt method was used to determine the differences between the target gene expression relative to the reference *Rp49* gene expression.

### Immunofluorescence

We performed immunostaining experiments for testis cells prepared using a protocol described previously (Tanabe et al., 2017). A pair of testes was collected from newly eclosed adult flies (within 2 days of eclosion). The fly was placed in a drop of the Testis buffer (183 mM KCl, 47 mM NaCl and 10 mM EDTA, pH 6.8) and was dissected under a stereomicroscope. Using a pair of forceps, the fly abdomen was clamped and the external genitalia were gently pulled outward. After carefully removing accessory tissues away from a pair of testes, the testes were collected on a glass slide. Using a pair of fine tungsten needles, the sheath covering the testis was torn from the apical tip and covered with an 18×18 mm coverslip (Matsunami, Osaka, Japan). After freezing the slides in liquid nitrogen, the coverslip was removed with a razor blade. The slides were transferred into 100% ethanol for 10 min to dehydrate and fix the sample. Subsequently, testis cells were fixed again with 3.7% formaldehyde for 7 min. The slides were permeabilised in PBST (PBS containing 0.01% Triton-X100) for 10 min and blocking with 10% normal goat serum in PBS (Wako, Osaka, Japan) for 30 min at room temperature. For immunostaining experiments, the following primary antibodies were used: mouse anti-γ Tubulin (1:200; GTU88; #T6557; Sigma-Aldrich, St Louis, MO, USA), rabbit anti-Orbit (1:200; Inoue et al., 2004), guinea pig anti-Asl (Novak et al., 2014; 1:800; a gift of Jordan Raff, University of Oxford, Oxford, UK), anti-Cp110 (1:200; Franz et al., 2013; a gift of Jordan Raff, University of Oxford, Oxford, UK) and anti-acetylated tubulin antibody (1:100; #6-11B-1; Sigma-Aldrich, St Louis, MO, USA). After incubating with the primary antibody overnight at 4°C, the samples were washed in PBS for 10 min and labelled with goat anti-mouse or goat anti-rabbit IgG (H+L) conjugated with Alexa Fluor 488, 594 or 647 (Invitrogen, Carlsbad, CA, USA). The secondary antibodies were used at a dilution of 1:400. After incubation for 2 h at room temperature, the slides were washed in PBS for 10 min and in pure water for 1 min. For DNA staining, we used VECTASHIELD mounting medium with 4′,6-diamidino-2-phenylindole (DAPI; #H-1200; Vector Laboratories, Burlingame, CA, USA) or Prolong Gold with DAPI (Invitrogen, Carlsbad, CA, USA). Imaging was performed on an Olympus IX81 fluorescence microscope (Olympus, Tokyo, Japan) outfitted with excitation and emission filter wheels (Olympus, Tokyo, Japan). Cells were imaged with 40× or 100× objective lenses. GFP and RFP fluorescence images were captured with a CCD camera (Hamamatsu Photonics, Shizuoka, Japan). Image acquisition was controlled through the Metamorph (Molecular Device, Sunnyvale, CA, USA) software package running on a PC. We also used Nikon N-SIM super-resolution microscopy (Nikon, Tokyo, Japan) to acquire the super-resolution microscope images of centrioles in premeiotic spermatocytes using an oil immersion objective lens (CFI SR Apochromat 100×, 1.49 NA; Nikon, Tokyo, Japan). Near-simultaneous GFP and/or RFP fluorescence images were captured with an EM-CCD camera (Hamamatsu, Photonics, Shizuoka, Japan). Image acquisition and quantification of the centriole length were performed through NIS-Elements (Nikon, Tokyo, Japan) software running on PC.

### Proximity Ligation Assay

The proximity ligation assay (PLA), which enables detection of protein interactions within a cell, was performed according to the Duolink kit method (Nacalai Inc., Kyoto, Japan) as described previously (Okazaki et al., 2020). We applied the PLA method to examine a close association between Cp110 and GFP–Orbit. For detection of the complexes, we used anti-Cp110 antibody (1:300; a gift from Jordan Raff) and anti-GFP antibody (1:200; A-6455; Thermo Fisher Scientific, Waltham, USA) to detect complexes containing Cp110 and Orbit. Samples were observed with a fluorescence microscope (IX81; Olympus, Tokyo, Japan). Image acquisition was controlled through the Metamorph software version 7.6 (Molecular Devices) and processed with ImageJ version 1.51 or Adobe Photoshop CS4.

## Supplementary Material

Supplementary information

Reviewer comments
