# Supplementary material for: Orbit/CLASP determines centriole length by antagonising Klp10A in Drosophila spermatocytes
Source: J Cell Sci. 2021 Mar 26;134(6):jcs251231. doi: 10.1242/jcs.251231 (PMC8015252; doi:10.1242/jcs.251231)
Supplement: Supplementary information [file joces-134-251231-s1.pdf]

Figure S1

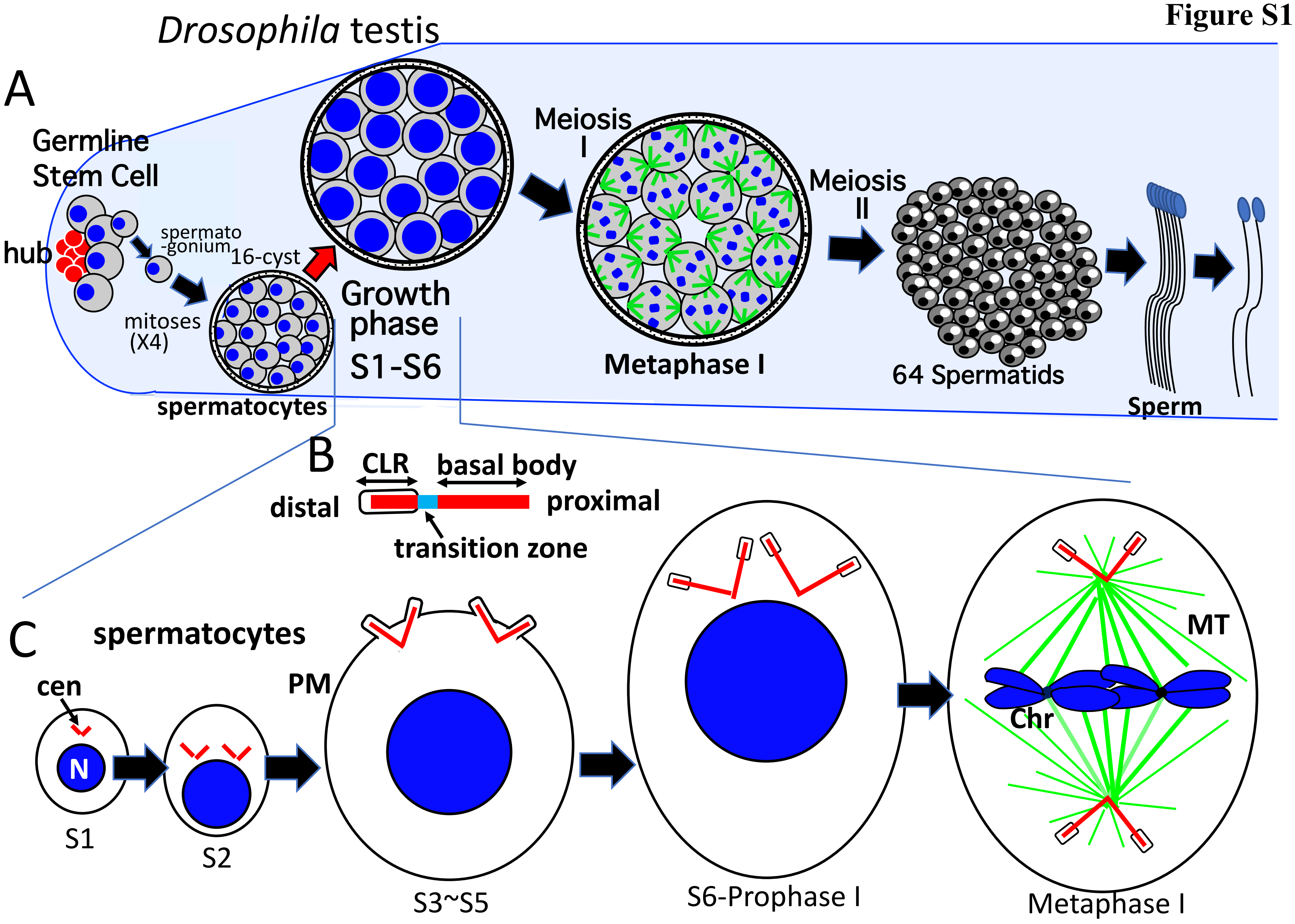

Fig. S1. Overview of cell growth and division in *Drosophila* spermatogenesis, and centriole dynamics in accordance with the cell growth of primary spermatocyte.
(A) Illustration of asymmetric division of germ line stem cells, four rounds of mitosis to generate the 16-cell cysts, cell growth of spermatocyte, and two meiotic divisions, meiosis I and II in *Drosophila* testis. (B) Schematic presentation of a single centriole consisted of the basal body, transition zone (light blue) and the ciliary-like region (CLR). (C) Dynamic alteration of a pair of centrioles during the growth phase, which is classified into S1 to S6 stages, and male meiosis I. Plasma membrane (PM), centriole (cen) (red), and microtubules (MT) (green). Nucleus (N) and chromosome (chr) are shown in dark blue.

Journal of Cell Science • Supplementary information

Figure S2

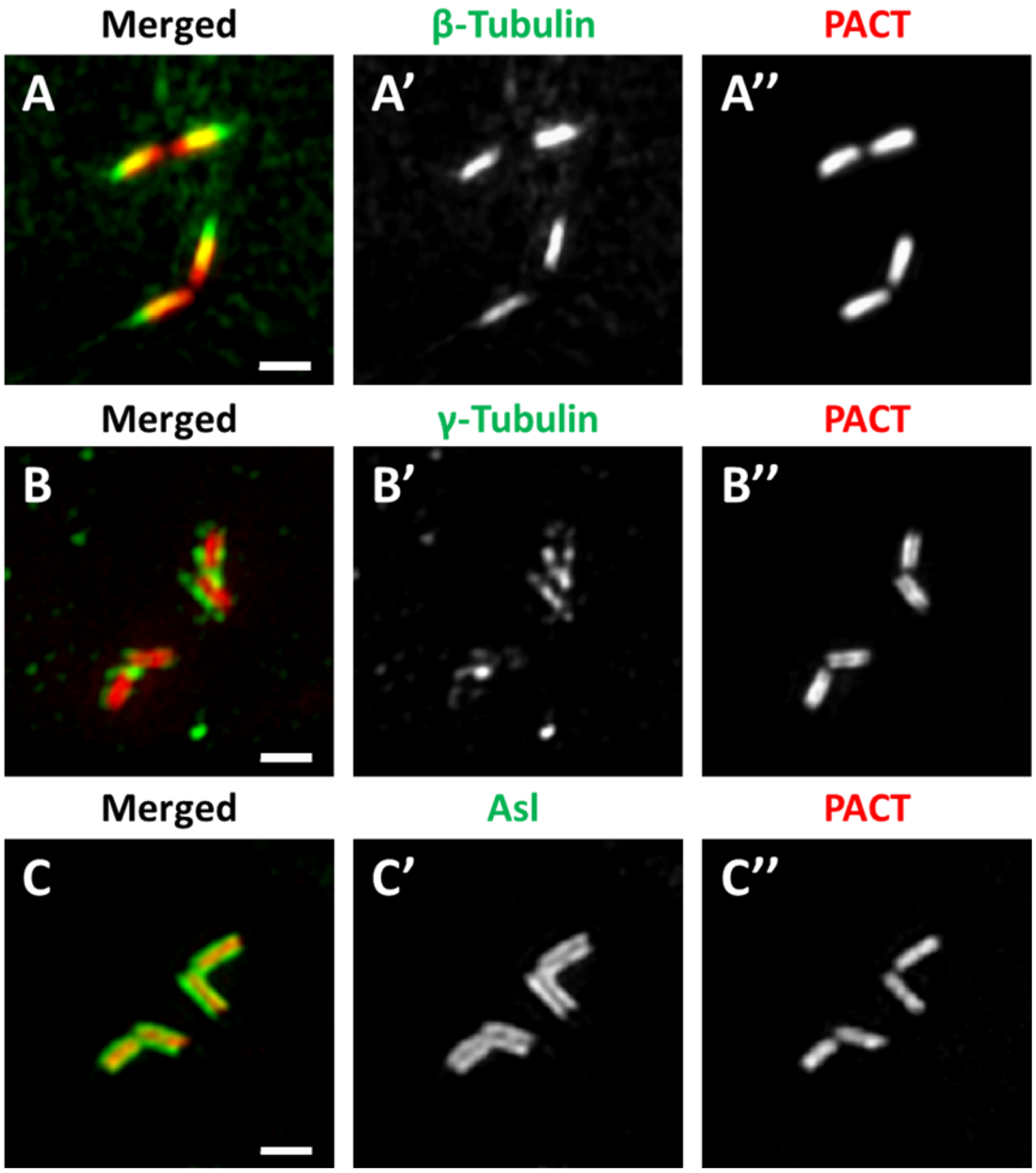

Fig. S2. Observation of two pairs of engaged centrioles by structured illumination super-resolution microscope (SIM), on which four types of centriolar proteins were distributed in premeiotic spermatocytes.

(A-C) Immunostaining of premeiotic spermatocytes expressing GFP-β-Tubulin or mRFP-PACT with the antibodies against the centriole-localized proteins, γ-Tubulin, and Asl. (A) Two pairs of centrioles in the premeiotic spermatocyte expressing GFP-β-Tubulin and mRFP-PACT. (A') GFP fluorescence of GFP-β-Tubulin on the centrioles. (A'') RFP fluorescence of mRFP-PACT on the of centrioles. (B and C) Immunostaining of two pairs of centrioles in the premeiotic spermatocyte expressing mRFP-PACT with anti-γ-Tubulin. (B') Anti-γ-Tubulin immunostaining of the centrioles. (C') Anti-Asl immunostaining of the centrioles. (B'' and C'') mRFP-PACT fluorescence of the centrioles. Note that γ-Tubulin and Asl are localized along outer region of the centriole cylinder. PACT and β-Tubulin are localized in lumen of the cylindrical structure of the centriole. The fluorescence images of the cells showing typical localization of the proteins were selected among more than 50 premeiotic spermatocytes for β-Tubulin and PACT fluorescence, 15 spermatocytes for γ-Tubulin and Asl immunostaining. bars: 1 µm.

Figure S3

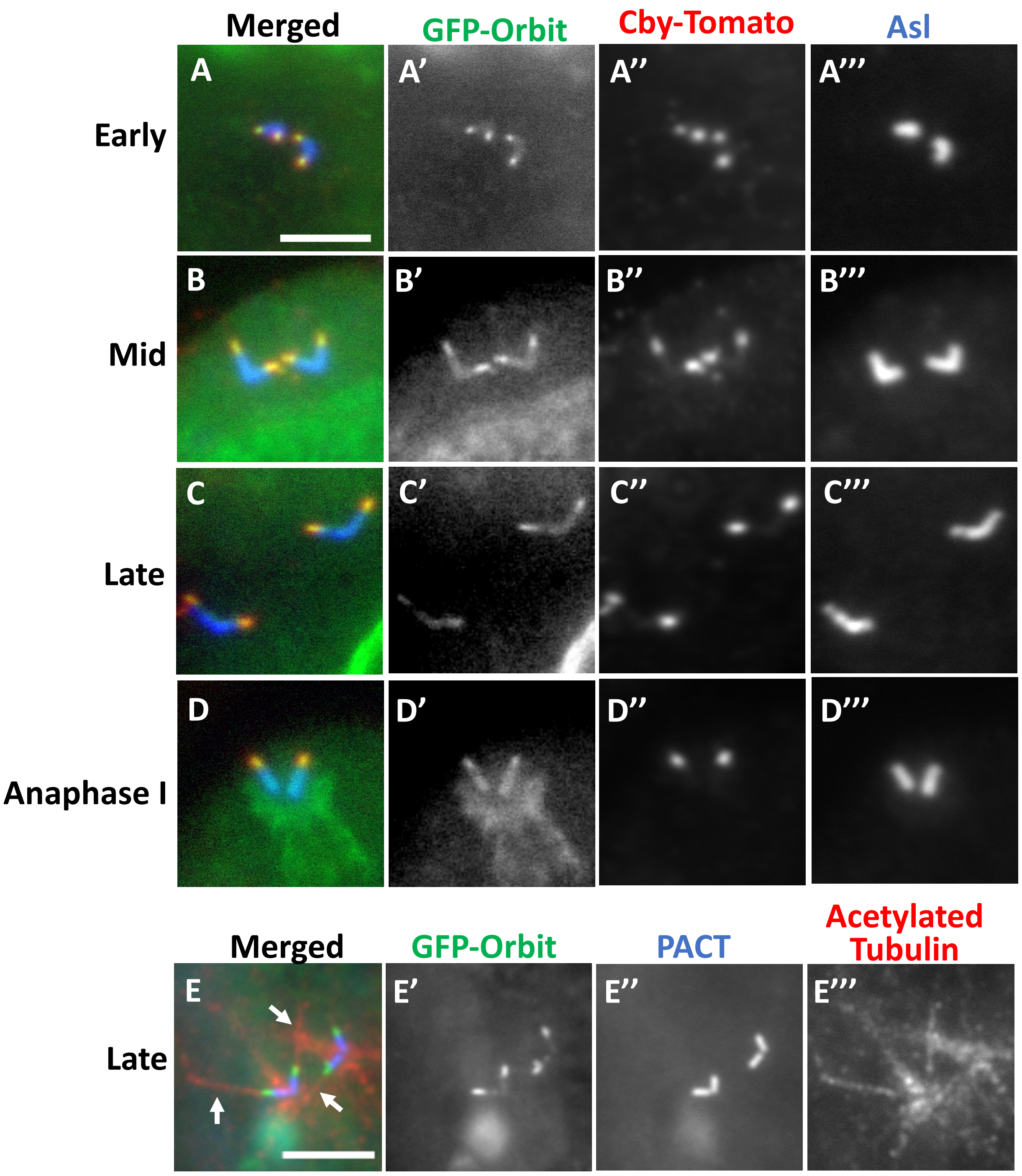

Fig. S3. Co-localization of Orbit with a transition protein, Cby and effect of Orbit overexpression on elongation of axoneme microtubules consisted of acetylated tubulin.
(A-D) Anti-Asl immunostaining of two sets of centrioles in premeiotic spermatocyte expressing GFP-Orbit and Cby-Tomato. Green in A-D (white in A'-D') represents a fluorescence of GFP-Orbit. Red in A-D (white in A"-D") represents a fluorescence of Cby-Tomato. Blue in A-D (white in A"'-D"') indicates anti-Asl immunostaining. GFP-Orbit is enriched at the distal region protruding from the distal end of basal body visualized by anti-Asl immunostaining. Cby-Tomato is localized on the most distal part of the Orbit-localizing region. Bar; 5μm. (E) Anti-acetylated tubulin immunostaining of two sets of centrioles in premeiotic spermatocyte expressing GFP-Orbit and mRFP-PACT. Green in E (white in E') represents a fluorescence of GFP-Orbit. Red in E (white in E"') represents axoneme microtubules recognized by anti-acetylated tubulin immunostaining. Blue in E (white in E") indicates mRFP-PACT fluorescence. Arrows indicate overly elongated axoneme microtubules from the distal ends of centrioles.

Journal of Cell Science • Supplementary information

Figure S4

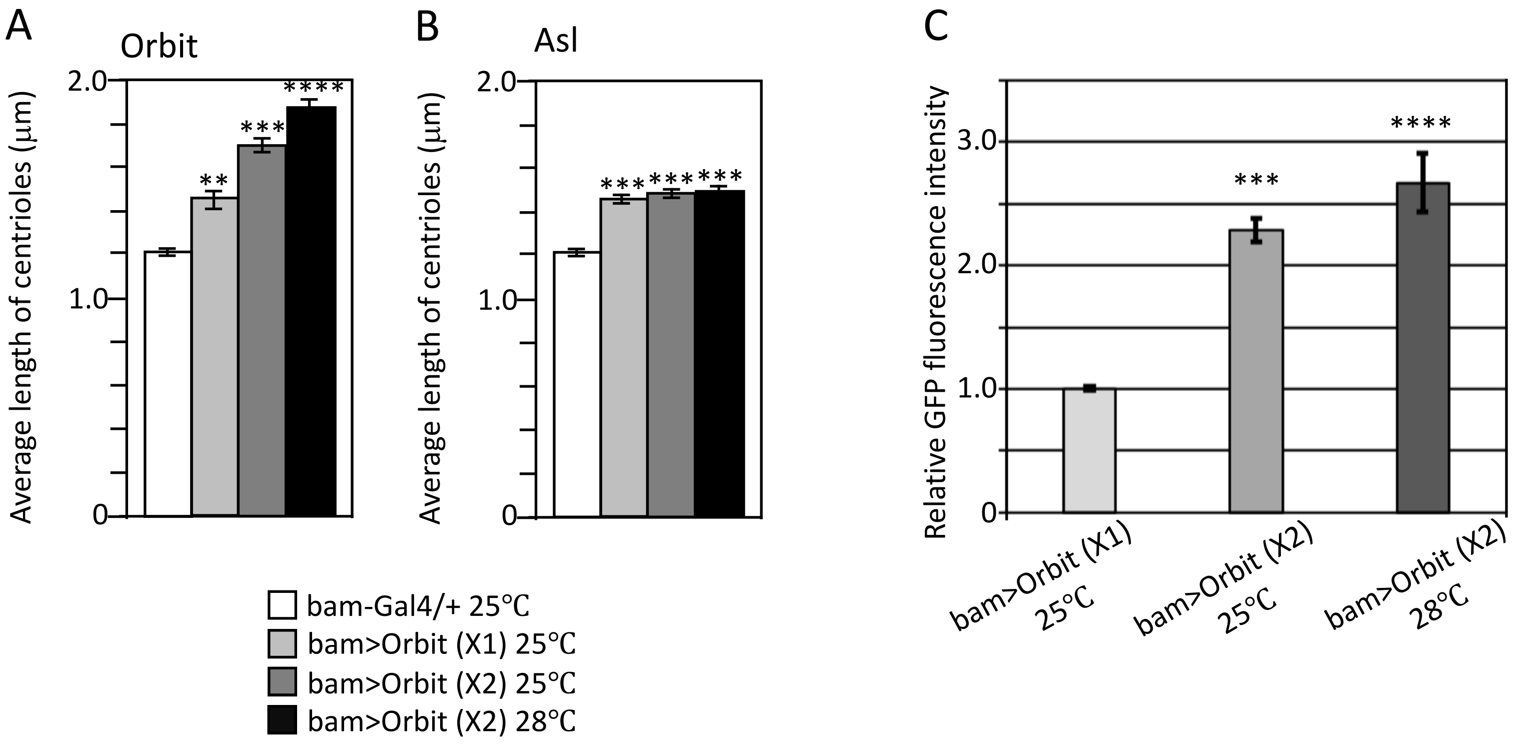

Fig. S4. Centrioles elongation is dependent on extent of Orbit overexpression.

(A, B) Average length of centrioles in control spermatocytes at S6 stage from control (*bam-Gal4/+*) males (while column), spermatocytes harbouring a single copy of *UAS-GFP-Orbit* and *bam-Gal4* (*bam>orbit*(X1)) from the males raised at 25℃ as lower level overexpression of the Orbit (light grey column), spermatocytes harbouring *bam-Gal4* and two copies of *UAS-GFP-Orbit* (*bam>orbit* (X2)) from the males raised at 25℃ as moderate level over expression (grey column), spermatocytes carrying *bam-Gal4* and two copies of *UAS-GFP-Orbit* (*bam>orbit* (X2)) from the males raised at 28℃ as higher level over expression (black column). (A) Average length of centrioles in control cells and the cells at same stage having Orbit overexpression at three different levels. Centriole length was measured by GFP-Orbit fluorescence. The length in control cells was measured by anti-Asl immunostaining. (B) Average length of centrioles in control cells and the cells having Orbit overexpression at three different levels. The length was measured by anti-Asl immunostaining. (C) Relative levels of GFP fluorescence intensity in the spermatocytes expressing GFP-Orbit at lower, moderate, or higher level overexpression. Total fluorescence intensity in whole regions of each cells was measured, and a relative intensity value was calculated, compared to that in the *bam>orbit*(X1) males. Charts show mean±S.E.M. Statistical significance was tested by Student's *t*-test, compared to average centriole length in control cells **; $P < 0.01$, ***; $P < 0.001$, and ****; $P < 0.0001$.

Journal of Cell Science • Supplementary information

Figure S5

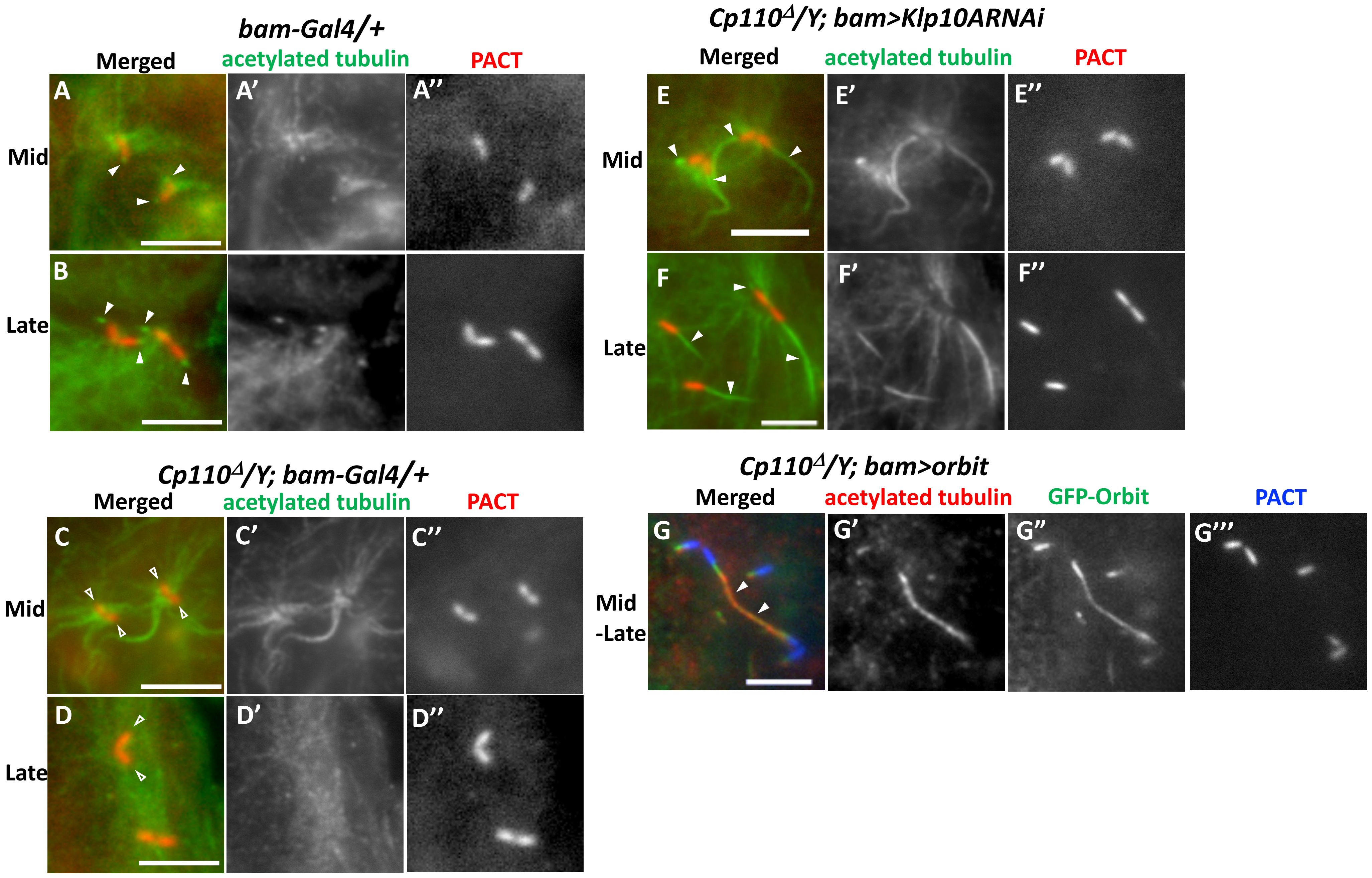

Fig. S5. Excess elongation of acetylated microtubules emanating from the distal ends of centrioles in *Cp110* null mutant spermatocytes overexpressing Orbit. (A-F) Anti-acetylated tubulin and anti-Asl immunostaining of spermatocytes expressing mRFP-PACT. (A, B) Control (*bam-Gal4/+*) spermatocytes at mid (A), and late (B) stages. (C, D) *Cp110* mutant (*$Cp110^{\Delta 1}$/Y;bam-Gal4/+*) spermatocytes at mid (C), and late (D) stages. (E, F) *Cp110* mutant spermatocytes harbouring *Klp10A* depletion at mid (E), and late (F) stages. (G) *Cp110* mutant spermatocytes overexpressing Orbit (*$Cp110^{\Delta 1}$/Y;bam>orbit*) at mid-late stage. Green in A-F (white in A’-F’) and red in G (white in G’) indicate anti-acetylated tubulin immunostaining. Red in A-F (white in A”-F”) and blue in G (white in G”’) indicate fluorescence of mRFP-PACT. Green in G (white in G”) indicates GFP-Orbit fluorescence. Note that microtubule caps composed of acetylated tubulin (arrowheads) exist at distal ends of centrioles visualised by PACT. The caps are absent at the distal ends in *$Cp110^{\Delta 1}$* mutant cells (open arrowheads). *$Cp110^{\Delta 1}$* spermatocytes having depletion of *Klp10A* or those having over-expression of *orbit* carry elongated acetylated microtubules from centrioles in both cases (arrowheads). Bars: 5 µm.

Figure S6

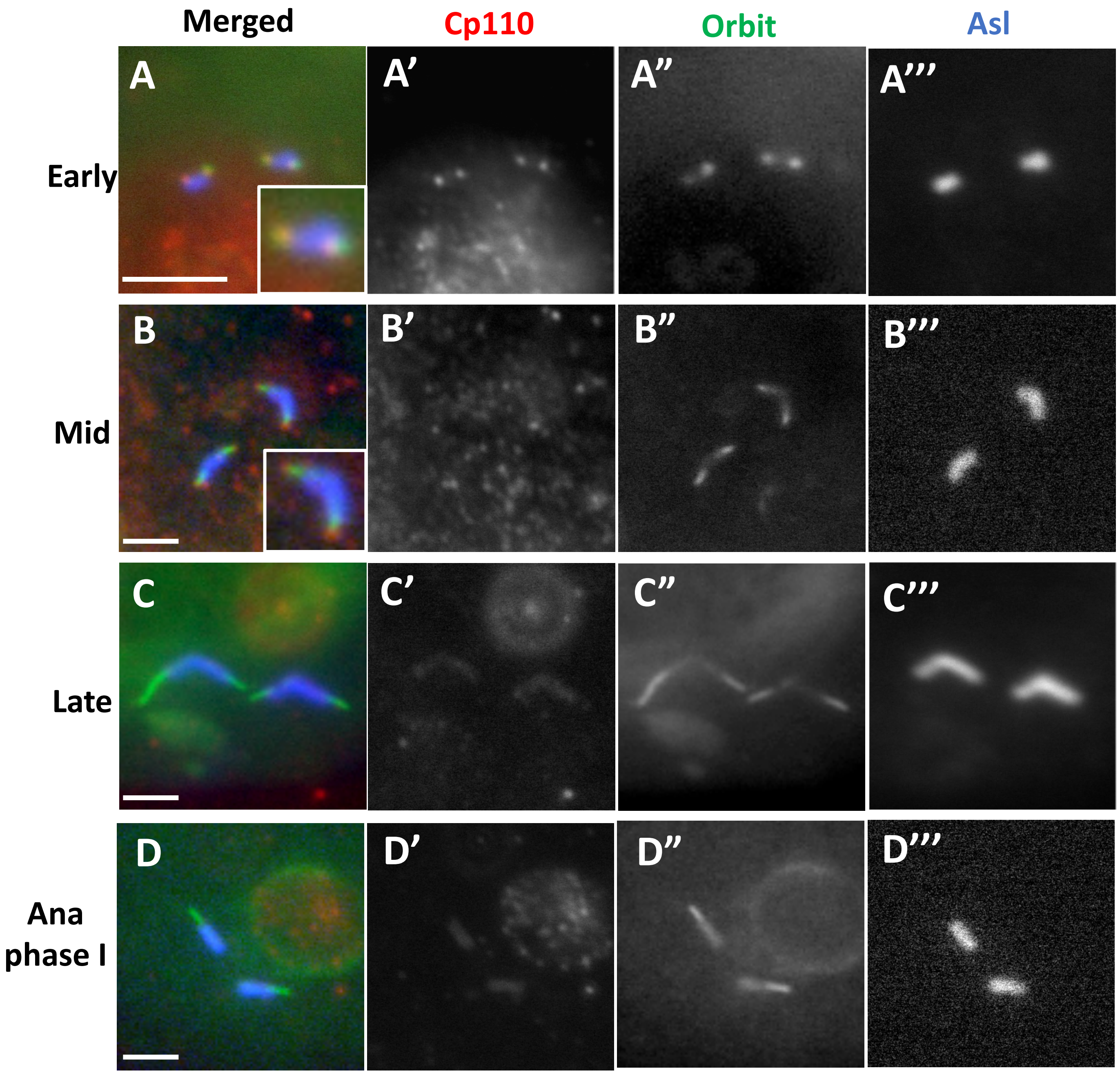

Fig. S6. The CP110 cap protein co-localized with Orbit at the distal ends of the centrioles in the earlier spermatocytes and disappeared from the ends at later stages.

Anti-CP110 and anti-Asl immunostaining of premeiotic spermatocytes expressing Orbit at early (A), mid (B), late stages (C), and meiotic stage (anaphase I) (D). Anti-Cp110 immunostaining (red in A-D, white in A’-D’), GFP-Orbit fluorescence (green in A-D, white in A”-D”), and anti-Asl immunostaining (blue in A-D, white in A”’-D”’). Bar, 1 µm. Magnified views of a set of centrioles in inset in A, and B. Orbit is enriched on the region extending from the distal end of basal body recognized by anti-Asl immunostaining. The Cp110 protein is localized on the distal tip of the Orbit-localizing region. The cap protein is diminished at the mid stage and fails to be observed at late stage and thereafter. Bars; 2µm.

Journal of Cell Science • Supplementary information

Figure S7

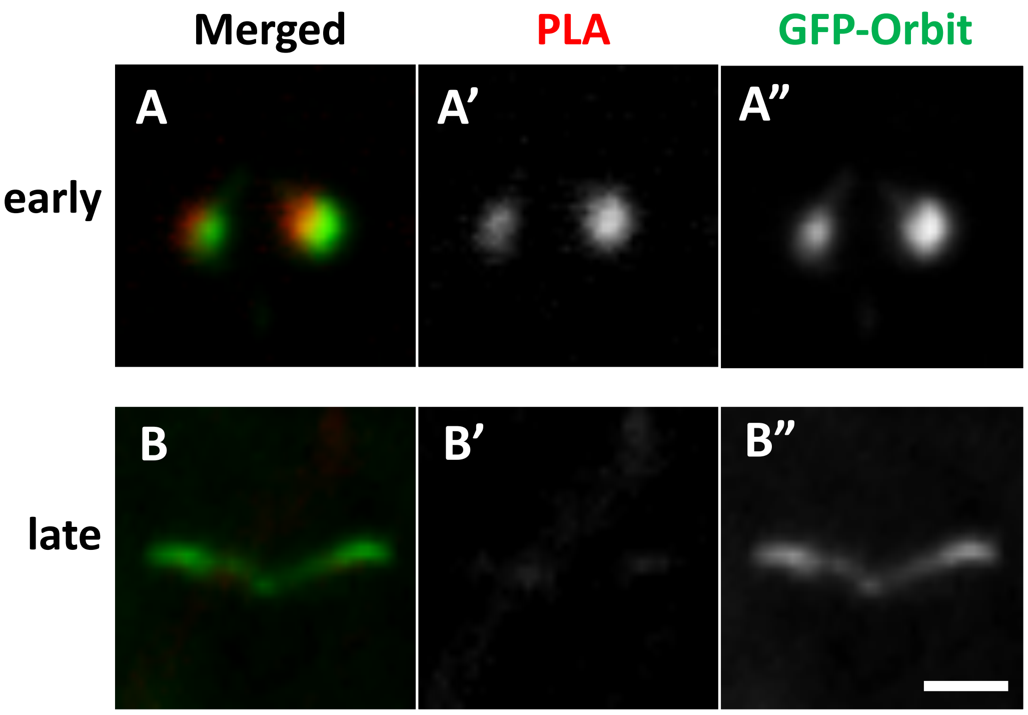

Fig. S7. Proximity Ligation Assay (PLA) to detect a complex containing Orbit and CP110. (A, B) PLA signal indicating that Orbit and CP110 were closely associated with each other within 40 nm in a pair of centrioles in early spermatocytes (A), but not in late spermatocytes (B). The PLA signals (Red in A, white in A') are observed on the one end of the centriole, while any PLA signals was not observed in B and B'. The GFP signals (Green in A and B, white in A" and B"). Bar, 1 µm.

Figure S8

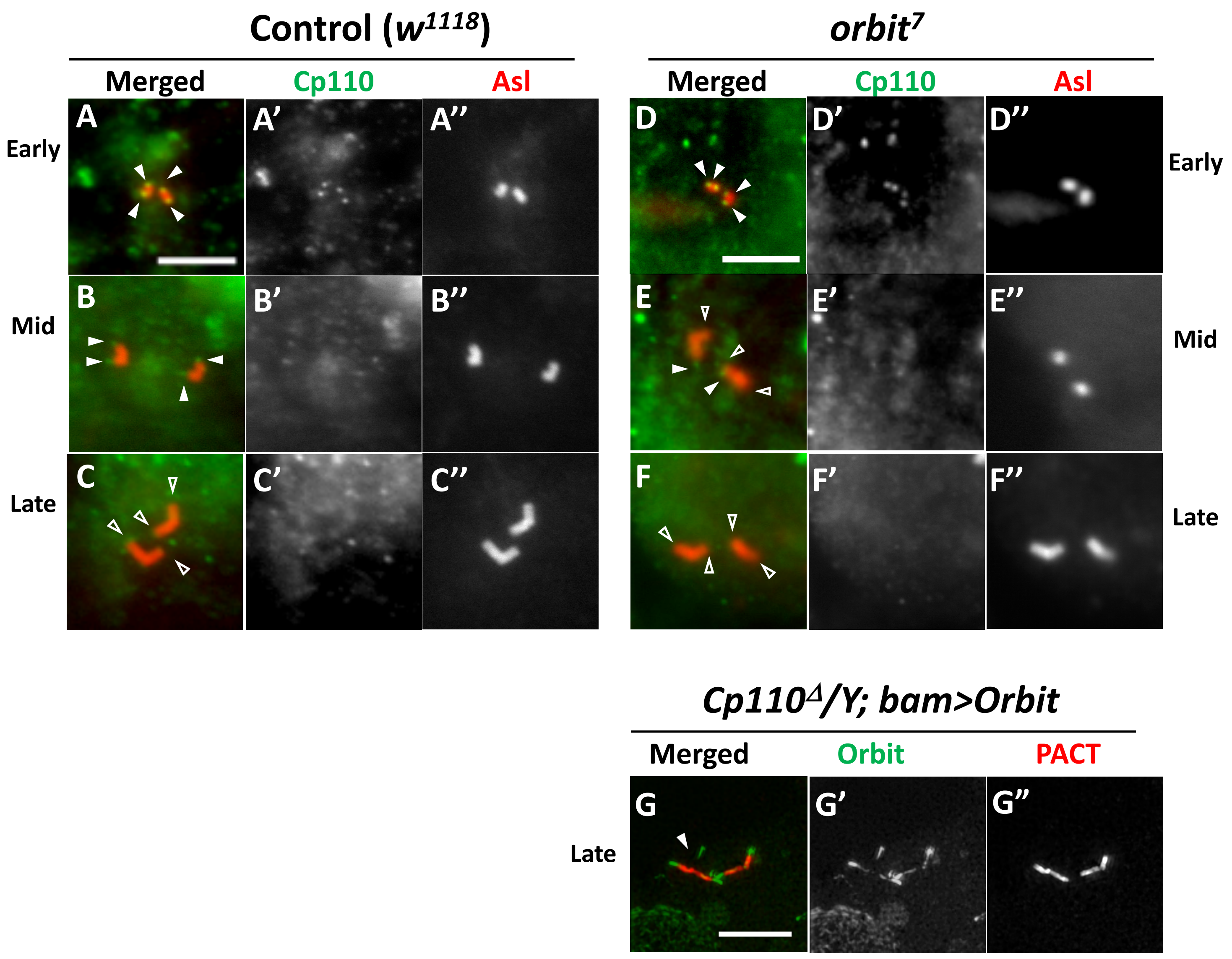

Fig. S8. A mutual dependence between Orbit and Cp110 on the localization on centrioles. (A-F) Immuno-localization of Cp110 on centrioles visualised by anti-Asl immunostaining in early, mid, and late stage spermatocytes from control (w^{1118}) (A-C), and *orbit*^{7} mutant males (D-F). Green in A-F (white in A’-F’) corresponds to anti-Cp110 immunostaining. Red in A-F (white in A”-F”) corresponds to anti-Asl immunostaining. Four Cp110 foci can be seen in every mutant spermatocyte among more than 50 earlier cells examined (filled arrowheads in A). The Cp110 foci become very faint at mid stage (arrowheads in B) or disappeared at late stage (open arrowheads in C). Consistently, the foci in *orbit*^{7} cells can be seen in early stage spermatid, thereafter they disappear as the growth phase proceeds (E, F), as shown in control cells (A-C). (G) A SIM observation of Orbit localization at the distal end of the basal body in a *Cp110*$^{\Delta 1}$ mutant spermatocyte. Green; GFP-Orbit, red; mRFP-PACT. Bars, 2 μm.

Journal of Cell Science • Supplementary information
