# Supplementary material for: Orbit/CLASP determines centriole length by antagonising Klp10A in Drosophila spermatocytes
Source: J Cell Sci. 2021 Mar 26;134(6):jcs251231. doi: 10.1242/jcs.251231 (PMC8015252; doi:10.1242/jcs.251231)
Supplement: Reviewer comments [file joces-134-251231_review_history.pdf]

Orbit/CLASP determines centriole length by antagonising Klp10A in *Drosophila* spermatocytes

Tsuyoshi Shoda, Kanta Yamazoe, Yuri Tanaka, Yuki Asano and Yoshihiro H. Inoue
DOI: 10.1242/jcs.251231

Editor: David Glover

Review timeline
Original submission: 3 July 2020
Editorial decision: 17 August 2020
First revision received: 19 January 2021
Accepted: 10 February 2021

Original submission

First decision letter

MS ID#: JOCES/2020/251231

MS TITLE: Orbit/CLASP determines centriole length by antagonising Klp10A in *Drosophila* spermatocytes

AUTHORS: Tsuyoshi Shoda, Yuki Asano, Yuri Tanaka, and Yoshihiro H. Inoue
ARTICLE TYPE: Research Article

We have now reached a decision on the above manuscript.

To see the reviewers' reports and a copy of this decision letter, please go to: https://submit-jcs.biologists.org and click on the 'Manuscripts with Decisions' queue in the Author Area. (Corresponding author only has access to reviews.)

As you will see, the reviewers raise a number of substantial criticisms that prevent me from accepting the paper at this stage. They suggest, however, that a revised version might prove acceptable, if you can address their concerns. If you think that you can deal satisfactorily with the criticisms on revision, I would be pleased to see a revised manuscript. We would then return it to the reviewers.

We are aware that you may currently be unable to access the lab to undertake experimental revisions. If it would be helpful, we encourage you to contact us to discuss your revision in greater detail. Please send us a point-by-point response indicating where you are able to address concerns raised (either experimentally or by changes to the text) and where you will not be able to do so within the normal timeframe of a revision. We will then provide further guidance. Please also note that we are happy to extend revision timeframes as necessary.

Please ensure that you clearly highlight all changes made in the revised manuscript. Please avoid using 'Tracked changes' in Word files as these are lost in PDF conversion.

I should be grateful if you would also provide a point-by-point response detailing how you have dealt with the points raised by the reviewers in the 'Response to Reviewers' box. Please attend to all of the reviewers' comments. If you do not agree with any of their criticisms or suggestions please explain clearly why this is so.

© 2021. Published by The Company of Biologists under the terms of the Creative Commons Attribution License (https://creativecommons.org/licenses/by/4.0/).

Reviewer 1

Advance summary and potential significance to field

The paper by Tsuyoshi Shoda et al. on “Orbit/CLASP determines centriole length by antagonising Klp10A in Drosphila spermatocytes” addressed the role of Orbit, Klp10A and CP110 in centriole elongation and engagement in drosophila spermatocytes. The authors nicely show that orbit antagonise Klp10A in the control of centriole length. They went on addressing the relationship of orbit with the centriolar distal complex CP110/ Cep97, already shown to participate in the regulation of centriole length. They additionally show that orbit overexpression has no additive effect with Cp110 deletion for centriole elongation but do not provide any further hypothesis on their relationship. Finally they suggest that CP110 cooperates with orbit and klp10A for centriole separation without assessing the mechanism of this control.
However all, the manuscript present nice data increasing our knowledge on the control of centriole elongation but the interpretation of the data is difficult and sometimes overstated. Thus, the manuscript is, in my opinion, not suitable as it stand for publication in JCS .

Comments for the author

Major points:
1) The data on centriole phenotypes are particularly difficult to interpret for several reasons:
- There are no methods to describe what was considered in the different populations for example does early separation include cells with several pieces of centrioles? How is made the difference between breakage and overduplication? How were quantified cells presenting two phenotypes?
- There is no evidence of overduplication or breakage. Small pieces could be pro-centrioles or broken pieces of centrioles, conversely overduplicated centrioles could be overly long centrioles broken in two pieces of approximately 1um each or bona fide centrioles. Super-resolution or EM data might help to decipher what is really observed.
- The analysis has been done on spermatocytes only, preventing the reader to assess whether centrioles were already perturbed before elongation proceeds.

2) The study on the role of the distal complex composed of CP110/ Cep97 in centriole elongation is incomplete.
- The fact that CP110 influences centriole length depending on the cell type and cellular context as already been shown in three independent studies (Delgehyr et al., 2012; Dobbelaere et al. 2020; Franz et al. 2013). A short discussion on the differences between the studies putting in light the differences in centriole structure (Jan et al. 2018) and tubulin modifications might help none drosophila’s users to understand why there might be differences.
- This study confirms that CP110 depletion increases centriole length in spermatocytes. However, the authors did not check whether indeed post-translational modifications of tubulins were affected by the depletion (Dobbelaere et al. 2020) and whether the other mutations (orbit, klp10A) affect the modifications.
- Cep97 mutants seem to have milder phenotypes than Dobbelaere et al. 2020. However, the authors use hypomorphic mutants that might still have proteins (according to flybase). The differences should at least be discussed or the data removed.
- The double mutant CP110Delta and orbit overexpression have similar centriole length than single mutant suggesting they are in the same pathway. Are they necessary for the localisation of each other or for tubulin post-translational modifications or for the recruitment of other stabilising factors such as Sas6?

3) Finally, there is no attempt to comprehend the centriole separation phenotype. Sas6 and Ana2 are really good candidates as they are involved in centriole engagement (Stevens et al. 2010) and centriole length regulation (Jana et al. 2018).

Minor points:
- A drawing explaining drosophila male gametogenesis and what the S1 to 6 stages are, when the transition zone and the primary cilia appear might help.
- Orbit localisation could be detailed during the time course of spermatogenesis and according to other markers like the distal CP110/ Cep97 complex and the transition zone.

© 2021. Published by The Company of Biologists under the terms of the Creative Commons Attribution License (https://creativecommons.org/licenses/by/4.0/).

- In orbit overexpressing condition, the authors should check with different staining that the PACT stains the entire length of centriole and whether the axoneme and the transition zone are perturbed.
- The authors should check for overstatement as they denote bias in the way the study was conducted. For example, the sentence: “the observation that Orbit localised around the distal plus end of centriole suggested that it was involved in centriole elongation”. This observation might indeed indicate that or a role in the transition zone, or in anchorage of the centrioles or many other things. There are many occurrences of such overstatement.
- I do not think it is reasonable in science to assume anything. So for the modulation of Orbit overexpression the authors should assess their statement either by quantitative immunofluorescence or by WB.
- It would be interesting to know what happens if both klp10A and orbit are depleted to see whether the antagonism explain all the phenotype observed in klp10A mutant.
- There is no graph for the centriole length of CP110Delta, orbit overexpression.
- The authors should present and attempt to understand the data on CP110 spindles. Indeed it is puzzling to think that they present “broken” centrioles (24%) that do not organise microtubules, on contrary of the other mutants.
- The authors should be careful in their conclusions as some of their mutants are not null (hypomorphic or RNAi).

<u>Reviewer 2</u>

Advance summary and potential significance to field

In this manuscript, Shoda et al characterise Orbit’s role, as a microtubule polymerizing factor, in the context of centriole elongation in Drosophila premeiotic spermatocytes. The authors begin by investigating the localisation of Orbit in the elongated centrioles of mature primary spermatocytes and find that it accumulates along the entire length of the centriole but is enriched in a region that extends beyond the distal end of the centriole. Spermatocyte-specific overexpression of Orbit shows an increase in centriole length, which is in fact dosage dependent, while hypomorphic mutants of orbit display shorter centrioles. The authors conducted similar overexpression/depletion studies of Klp10A and reported findings in agreement with a previous study (Delgehyr et al. 2012). Orbit and Klp10A have previously been reported to have antagonistic roles in regulating spindle length (Laycock et al. 2006). Thus, Shoda et al investigated if a similar mechanism regulated centriole length as well. The simultaneous overexpression of Orbit and RNAi-mediated depletion of Klp10A resulted in enhanced centriole elongation that lead to additional defects associated with extremely long centrioles. Moreover, combining Orbit overexpression/Klp10A depletion individually with a null mutant of CP110, a component of the distal tip complex, resulted in similar severe phenotypes associated with extremely long centrioles. Taken together, the authors concluded that excess centriole elongation in the absence of the distal tip complex that functions as a protective lid results in loss of centriole integrity.

The mechanism of centriole elongation in Drosophila remains elusive with only a few studies that have attempted to address this question. Studies of Klp10A have established that it is an important regulator of centriole length in Drosophila. Given that centriole length is believed to be regulated as a balance between polymerisation and depolymerisation of centriolar microtubules this manuscript’s focus on Orbit’s role as a polymerisation factor complements studies of Klp10A. Therefore, the manuscript would be of interest to the field and I support its publication. However, the manuscript needs major revision.

Comments for the author

Major points:
1.Fig S1A – Do all centrioles marked with β-tubulin and PACT show a similar shift in signal? It would a be good to include a sentence about your hypothesis on why such a distribution is observed.

© 2021. Published by The Company of Biologists under the terms of the Creative Commons Attribution License (https://creativecommons.org/licenses/by/4.0/).

2.Fig 2A/A' – Orbit signal along the length of the centriole is not visible in the image provided. It appears as if Orbit is localised only in the region beyond the distal end of the centriole. An image like in Fig 2B will be appropriate.

3.Fig S2A – The graphs do not match the description provided in the figure legend. The bar graph of the control is missing. If centriole length was measured with GFP-Orbit signal, how were the control centrioles measured in the absence of a GFP-Orbit transgene?

4.Despite centriole elongation, 100% of the centrioles were engaged in Orbit overexpression. For Klp10A RNAi, besides centriole elongation, were there any other phenotypes associated with defects in centriole integrity because of the long centrioles? If so, please do present the data in Figure 4.

5.Fig 5A – Marker used for the centriole length measurements is not indicated in the figure legend.

6.Fig 5B-E – GFP-Orbit signal looks very elongated in these centrioles. It will be nice to have a brief description about this phenotype in the results section in addition to the centriole elongation and loss of centriole integrity phenotype – why does the signal appear like this/your hypothesis.

7.Lines 294-304 describe the phenotypes observed in CP110 mutants. However, the data has not been presented in the main/supplementary figures. Please include the data.

8.For Figs 6A-H, for each of the genotypes, please briefly mention in the results section the effect on centriole length - whether it is shorter/longer than in the control in each scenerio.

9.For clarity, it will be better to briefly describe why no centriole integrity loss phenotypes were observed for Klp10A overexpression in CP110 mutant background and Klp10 overexpression spermatocytes.

10. Data for control and Cp110 mutant bipolar meiotic spindle is not shown in Fig 7. Please include the data.

Minor Points:

1.Fig 4F/F' – There appears to be a black box in the figure that is visible in the single DNA channel.

2.Line 233 – "Toward this purpose, we overexpressed Orbit and Klp10A simultaneously in.." 'Depleted' is missing in front of Klp10A.

3.Fig 6B – PAC should be PACT.

4.Line 277 – Citation should be (Galletta et al. 2016) instead of 2010.

5.A recent study on Cep97 - 'Cep97 Is Required for Centriole Structural Integrity and Cilia Formation in Drosophila' (Dobbelaere et al. 2020) discusses its role in regulation of centriole length. It may be good to briefly discuss the centriole elongation findings of the Cep97 paper in the Discussion section to complement the findings of CP110 presented in this paper.

<u>Reviewer 3</u>

Advance summary and potential significance to field

In this manuscript the authors used the versatility of Drosophila genetics and cell biology to demonstrate that the microtubule polymerizing factor Orbit/CLASP contributes to the determination of centriole length and antagonizes Klp10A. Moreover, the authors bring into the picture the CP110 and the distal tip complex and also used the very reliable genetic interactions to demonstrate how this complex interacts with Orbit and Klp10A to ensure proper centriole lenght. In addition to the significance of these results giving to hot centriole elongation field, it also has relevance in cancer research demonstrating that centriole over-elongation by disturbance of these

© 2021. Published by The Company of Biologists under the terms of the Creative Commons Attribution License (https://creativecommons.org/licenses/by/4.0/).

factors leads to loss of centriole integrity, consequently multipolar spindle thus chromosomal instability.
This reviewer does encourage the acceptance and publication of this paper and has only few suggestion to the authors with the intention to help to improve the quality of the manuscript.

Comments for the author

line 32: "physically" - the only evidence for physical interaction is the proximity ligation assay which "suggest" but does not convincingly prove a physical interaction. Someone would expect here in-vitro binding assays, Co-IP-s to this end. Therefore I suggest removing this word.

line 55: sentence "The C-terminal domain of Dplp (a PCM protein), called the pericentrin-AKAP450 centrosomal targeting domain (PACT), also localises along the centrioles." These sentence in the introduction break the nice line of thought of the text. I understand that the authors wish to highlight PACT since they will use this later as a marker based on which they measure the centriole length. I suggest to put this reference sentence in the result section after line 160.

line 175: just the localization on its own doesn't raise the possibility. I suggest rephrasing: "localization together with the known Mt polymerization role suggest..."

line 206: please indicate in a half-sentence you used hypomorph orbit allele (a reader interested in Drosophila genetics might want to know why only this)? Was null not available? Is the orbit null lethal in early developmental stage? Using hypomorph raises the possibility that we don't see the maximum of the effect of orbit loss, but it is not a problem since still demonstrate the function. I'm just curious if it is known the phenotype of an orbit null. And if yes, would be nice to mention here.

line 218: very good that authors coexpressed dicer2 since the adults fly doesn't express well Dicer2 and that diminishes the efficiency of RNAi thus dcr2 coexpression is needed. A reader with little fly genetics background might wonder what does UAS-dcr2 mean, so it would be great to mention in a sentence in the results or in the figure legend and explain well in Materials and Methods.

line 233: Please correct this sentence. It is obvious from the given genotype that it is not simultaneous overexpression of Orbit and Klp10A but overexpression of orbit combined with Klp10A knockdown.

There is a typo on Figure 6 C and D, "dcr2" is mistyped as "dsr2".

First revision

Author response to reviewers' comments

Reviewer 1 Comments for the Author:

Major points:
1) The data on centriole phenotypes are particularly difficult to interpret for several reasons:
①There are no methods to describe what was considered in the different populations for example does early separation include cells with several pieces of centrioles? How is made the difference between breakage and overduplication? How were quantified cells presenting two phenotypes?
i)Does early separation include cells with several pieces of centrioles?

（Response）

Taking the reviewer’s comment into account, we added sentences to explain methods by which we classified abnormal centrioles in Results. We classified the abnormal centrioles to three classes, disengagement (revised from “early separation”), breakage, and overduplication. (line 270-285, page 14- 15).

© 2021. Published by The Company of Biologists under the terms of the Creative Commons Attribution License (https://creativecommons.org/licenses/by/4.0/).

"We classified the abnormal centrioles to three classes, disengagement, breakage, and overduplication. When we observed abnormal cell with two or four unpaired centrioles of full-length (approximately 1μm or longer), we considered that disengagement of either or both pairs of centrioles occurred precociously before meiosis. Thus, we categorized the cell as disengagement. If we found a cell with unpaired centriole pieces shorter than 1□m, we classified it as breakage, rather than disengagement. A cell carrying extra pairs or single full-length centrioles were classified as overduplication. Shorter centrioles in *orbit*[7] mutant cells and *bam>Klp10A* cells were not included in the loss of integrity phenotypes, as far as they were engaged. In addition, we have observed spermatocytes showing two types of phenotypes at the same time. For example, we found the cells showing both disengagement and breakage phenotypes (Fig. 5E). To avoid counting these cells twice, we categorized them as breakage. When we have categorized the cell showing both breakage and overduplication, we categorized it as overduplication."

②*There is no evidence of overduplication or breakage. Small pieces could be pro-centrioles or broken pieces of centrioles, conversely overduplicated centrioles could be overly long centrioles broken in two pieces of approximately 1um each or bona fide centrioles. Super-resolution or EM data might help to decipher what is really observed.*

(Response)

We presented representative super-resolution microscopy (SIM) images of abnormal centrioles observed in spermatocytes having *orbit* overexpression and/or *klp10A* depletion in Fig. 2-7 and Fig. S2, S8G and S9B,C. We classified the abnormal figures into three classes as shown in Fig. 5C-F, Fig. 7B-F and 7I. As the reviewer pointed, small PACT-positive pieces could be disengaged forms of pro-centrioles, broken pieces of centrioles, or bona fide centrioles of approximately 1 micrometer long. If the pieces were disengaged, they were considered as disintegrated phenotypes and classified into disengaged or breakage, according to whether they are shorter than 1micrometer long or not. If the pieces were engaged, it is more difficult to conclude that the pieces were normal proto-centrioles or broken pieces. Actually, we have never see such a phenotype in normal cells. Therefore, it is unlikely that they are normal proto- centrioles.

③*The analysis has been done on spermatocytes only, preventing the reader to assess whether centrioles were already perturbed before elongation proceeds.*

(Response)

It is another interesting issue to investigate whether Orbit and Klp10A are required for centriole elongation in spermatogonia, besides spermatocytes before meiosis. For overexpression or depletion of Orbit and Klp10A, we used a *bam-Gal4* driver that can induce UAS-dependent gene expression in both spermatogonia and spermatocytes. In theory, we might be able to detect a alteration in the length in spermatogonia, if any. However, it was technically difficult to detect a subtle difference in the cells from *orbit* mutant males or the cells overexpressing Klp10A even under a super-resolution microscope. We will attempt to address the issue using a transmission electron microscopy or a better high-resolution microscopy in near future. Therefore, we are not able to exclude a possibility that centrioles had already been perturbed before they begun to elongate in the growth phase of spermatocytes.

2) The study on the role of the distal complex composed of CP110/ Cep97 in centriole elongation is incomplete.

①*The fact that CP110 influences centriole length depending on the cell type and cellular context as already been shown in three independent studies (Delgehyr et al., 2012; Dobbelaere et al. 2020; Franz et al. 2013). A short discussion on the differences between the studies putting in light the differences in centriole structure (Jan et al. 2018) in tubulin modifications*

(Response)

According to the reviewer's comment, we added the following sentences in Discussion (line 475, page 24). "The fact that Cp110 influences centriole length depending on the cell type and cellular context as shown in previous studies (Delgehyr *et al.*, 2012; Dobbelaere *et al.* 2020; Franz *et al.* 2013). It is likely that differential regulation of the conserved core components underlies ciliary basal diversity in different cell types. As argued previously(Jana *et al.*, 2018), cellular-specific and tissue specific regulation in centriole duplication may be indispensable to regenerate diverse

© 2021. Published by The Company of Biologists under the terms of the Creative Commons Attribution License (https://creativecommons.org/licenses/by/4.0/).

centriole structure."

In this manuscript, we focus on the mechanism by which centrioles with a certain length are produced, rather than on the role of the distal complex containing CP110 in centriole organization. However, considering the reviewer's valuable comment, we added several sentences mentioned above in Discussion.

②*This study confirms that CP110 depletion increases centriole length in spermatocytes. However, the authors did not check whether indeed post-translational modifications of tubulins were affected by the depletion (Dobbelaere et al. 2020) and whether the other mutations (orbit, klp10A) affect the modifications.*
(Response)

According to the reviewer's comment, we performed anti-acetylated tubulin immunostaining to examine whether the stabilized microtubules were present at the distal tip of the centriolar microtubules in the absence of Cp110, and whether Orbit overexpression or Klp10A depletion affects the tubulin modification in the *Cp110* mutant cells. We added the following sentences and Fig. S5 in Results (line 378, page 19). " Moreover, we investigated whether post-translational modifications of tubulins were indeed affected by depletion of the *Cp110* gene, or the null mutation. In the absence of another component of the distal end complex, Cep97, tubulin acetylation was inhibited (Dobbelaere *et al*. 2020). Acetylated tubulin foci appeared on the distal ends of centrioles in control spermatocytes at mid stage (Fig. S5A) and became elongated at late stage (Fig. S5B). The foci failed to be observed at the distal ends in the *Cp110* null mutant cells (Fig. S5C, D). These observations were consistent with the published results in the *Cep97* null mutants (Dobbelaere *et al*. 2020). Furthermore, we examined whether Orbit overexpression or Klp10A depletion influences tubulin acetylation in the absence of Cp110. Interestingly, we found that acetylated microtubules excessively grew from the distal ends in both cases, indicating that modification and elongation of the stabilized microtubules was not perturbed in altered expression of Orbit or Klp10A without Cp110. Rather, these genetic alterations resulted in enhanced elongation of axonemal microtubules."

③*Cep97 mutants seem to have milder phenotypes than Dobbelaere et al. 2020. However, the authors use hypomorphic mutants that might still have proteins (according to flybase). The differences should at least be discussed or the data removed.*
(Response)

For observation of centriole structure in *Cep97* mutant spermatocytes, we used $Cep97^{LL}$ mutation, which is a hypomorphic allele induced by PB element into the first intron of the gene. We have observed precocious disengagement of centriole pairs in all spermatocytes examined in the mutant males before meiosis. In contrast, Dobbelaere and colleagues have recently isolated a deletion mutant for *Cep97* gene and the authors described only excess elongation in the mutant spermatocytes (Dobbelaere et al. 2020). They did not described any other phenotypes in centriole organization in the mutant cells. As the null mutant was not available to us, we have not confirmed the phenotype. We do not know reason(s) why the hypomorphic mutant spermatocytes showed more sever phenotypes. We observed the same or even more sever phenotype in the hemizygous mutant cells over *Df(2L)ed* deficiency uncovering the gene.
However, to clarify the differences in phenotypes between the null and the hypomorphic mutants, we need more experiments. To avoid exceed of a word limit of the journal, we decided to remove all data regarding Cep97 and to put them in our next manuscript.

④ *The double mutant CP110Delta and orbit overexpression have similar centriole length than single mutant suggesting they are in the same pathway. Are they necessary for the localisation of each other* ***or*** *for tubulin post-translational modifications or for the recruitment of other stabilizing factors such as Sas6?*
(Response)

We would like to acknowledge the reviewer's valuable suggestions. According to the reviewer's comment, we performed Cp110 immuno-localization experiments in *orbit* mutant cells and vice versa. We added Fig. S8, the relevant figure legend, and the following sentences in Results (line 309,

© 2021. Published by The Company of Biologists under the terms of the Creative Commons Attribution License (https://creativecommons.org/licenses/by/4.0/).

page 16).
"Thus, we next investigated whether these proteins are interdependent on each other in centriole localization. We first performed anti-Cp110 immunostaining of control (Fig. S8A-C) and *orbit*[7] mutant spermatocytes (Fig. S8D-F) during spermatocyte development. The Cp110 was localized at the distal ends of centrioles in control spermatocytes at the early and mid-stages (75/84, 89.3% of the cells, no signals in 10.7% (9/84)). In the hypomorphic mutant cells at the same stages, the protein was comparably observed in 82.7% of the centrioles (91/110) (no signals in 17.3% (19/110)). On the contrary, we investigated whether Orbit is localized at the distal centriole ends in the spermatocytes from *Cp110* null mutant males. Among 66 centrioles in control spermatocytes expressing GFP-Orbit, the signal was present at distal ends on 97.0% (64/66) of centrioles (no signal in 3 % (2/66)). Similarly, among 85 centrioles in spermatocytes at mid and late stages from *Cp110* null mutant with GFP-Orbit expression, the signal was still localized at distal ends on 98.8% (84/85) of the centrioles (1.1 % (1/85) possessed no signal at either end of two pairs) (Fig. S8G). Therefore, we speculate that Orbit and Cp110 are not required for each other in mutual localization on centrioles."

To answer another inquiry "*Are they necessary for tubulin post-translational modifications*", we performed additional experiments to investigate tubulin acetylation of centriole microtubules in relevant mutants, and added figure S5 to demonstrate the results and the following sentences in Results (line 385, page 20): "Furthermore, we examined whether Orbit overexpression or Klp10A depletion influences tubulin acetylation in the absence of Cp110. Interestingly, we found that acetylated microtubules excessively grew from the distal ends in both cases, indicating that modification and elongation of the stabilized microtubules was not perturbed in altered expression of Orbit or Klp10A without Cp110. Rather, these genetic alterations resulted in enhanced elongation of axonemal microtubules."

We agree that it is also interesting to investigate whether other centriole elongation factors such as Sas6 are involved in the excessive elongation of the centriole microtubules in the spermatocytes having Orbit overexpression or Klp10A depletion in absence of Cp110. As Sas6 and Ana2 are known to be required for restriction of the centriole elongation in cooperation with Cp110 (Franz et al., 2013), it is possible to test whether the tubulin acetylation in the *Cp110* null mutant cells having Orbit overexpression or Klp10A depletion can be influenced in Sas6 mutant background. As we have no *Sas6* mutants or UAS-Sas6RNAi stocks, or antibody against Sas6 at the moment, we would like to address the issue in our next study. We appreciate the reviewer's fruitful comments.

3) Finally, there is no attempt to comprehend the centriole separation phenotype. Sas6 and Ana2 are really good candidates as they are involved in centriole engagement (Stevens et al. 2010).
(Response)
According to the reviewer's request, we added a separate paragraph describing our hypothesis on the mechanisms why a pair of centrioles separated earlier in *Cp110* mutant spermatocytes having Orbit overexpression and/or Klp10A depletion in Discussion (line 508-521, page 26). We appreciate the reviewer's fruitful comment.

"In addition to excessively elongated centrioles, we observed several abnormalities in centriole organisation and structure in spermatocytes overexpressing Orbit and/or harbouring *Klp10A* depletion. In the absence of Cp110, the loss of centriole integrity phenotypes was also enhanced. Small pieces of centrioles observed may be broken pieces of over-elongated centrioles, as observed in cancer cells (Marteil et al., 2018). Alternatively, they may have been unpaired centrioles separated precociously from centriole pairs containing daughter procentrioles, which are smaller than normal centrioles (Karki et al., 2017). By contrast, the loss of centriole integrity phenotypes was not observed in the cells overexpressing a shortening factor, Klp10A. Hence, we consider that improvised construction of the basal body microtubules may be associated with the loss of integrity phenotype. And thereby, centriole engagement would be lost. We found centriole fragments with shorter diameter in the centriole microtubules. The presence of disintegrated centrioles supports this idea. Further investigations are necessary to test the current hypothesis."

"Spermatocytes homozygous for a loss of function mutation of *Sas6* and that of *Ana2* commonly demonstrated premature centriole separation before meiosis (Stevens *et al.*, 2010, Rodrigues-Martins *et al.*, 2007, Lattao *et al.*, 2017). Hence, Sas6 and Ana2 considered to be required for centriole engagement and/or maintenance of the pairs. Orbit overexpression and/or Klp10A

© 2021. Published by The Company of Biologists under the terms of the Creative Commons Attribution License (https://creativecommons.org/licenses/by/4.0/).

depletion may affect centriole engagement through interfering the Sas6 (or Ana2) function. It is also possible to consider that the premature disengagement can occur independent on Sas-6 or Ana2. Besides, it was reported that APC/C activation, and activation of separate, thereby unexpected cleavage of Scc1 cohesin can take place in mammalian cultured cells (Karki *et al.*, 2016). We cannot exclude a possibility that alteration of microtubule dynamics in centrioles by altered expression of Orbit and/or Klp10A led to unexpected APC/C activation. This hypothesis would be tested by several experiments in our next study."

Sas6 and Ana2 are really good candidates as they are involved in centriole length regulation (Jana et al. 2018).
(Response)
We would like to acknowledge the reviewer's valuable suggestion that Sas6 and Ana2 possibly interact with Orbit and/or Klp10A in centriole length regulation. We are considering the following hypothesis; In addition to construction of cartwheel components essential for centriole engagement at proximal ends, Sas6 and Ana2 are also required for elongation of centriole basal body through recruitment of Bld10. The microtubule binding protein can stimulate polymerization of central centriole microtubules (Carvalho- Santos, *et al*., 2012, Jana *et al*., 2018). Sas6 appeared to be localized along central singlet microtubule and play a role to regulate length of the centriole microtubules at distal end through interaction with Cp110 (Franz *et al*., 2013). Ana2 is required for recruitment of Sas6 onto centrioles in *Drosophila* cultured cells (Dzhindzhev *et al*., 2014). Hence, it is possible to speculate that overexpression of the microtubule polymerization factor and/or down-regulation of the shortening factor may affect localisation or function of Bld10, directly or via interaction with Sas6 and Ana2 at the distal ends of centriolar microtubules. As Orbit is distributed along centriole basal body, the protein and/or Klp10A can also interact with Sas6 and Ana2. Unfortunately, to avoid exceed of the word limit, we could not include it in this manuscript.

Minor points:

1 A drawing explaining drosophila male gametogenesis and what the S1 to 6 stages are, when the transition zone and the primary cilia appear might help.
(Response)

According to the reviewer's request, we added a drawing to explain *Drosophila* male gametogenesis (Figure S1A), and alteration of two pairs of centrioles in the growth phase of spermatocytes, which can be classified into different stages designated as S1 to S6 stages (Figure S1A, C), and organization of a centriole consisted of the cilium-like region, the transition zone and bosal body regions (Figure S1B).

2 Orbit localisation could be detailed during the time course of spermatogenesis and according to other markers like the distal CP110/ Cep97 complex and the transition zone.
(Response)

According to the reviewer's request, we examined Orbit localization during the time course of spermatogenesis while comparing with Cp110 which is a component of the distal complex or a transition zone protein, Chibby. We added the following sentences regarding co-localization of Orbit with Cp110 and Fig. S6 in line 296 page 16;
"First, we confirmed that Cp110 was localised on centrioles at the distal end in the earlier stages of premeiotic spermatocytes, but not at later stages (Fig. S6A-D). Orbit co-localized with Cp110 on distal tips of centriole basal body at early stage (Fig. S6A). Until mid-stage, Cp110 was localized on the most distal area of the Orbit-localizing region (Fig. S6B). The GFP-Orbit signal protruding from the distal end of centrioles overlapped with anti-acetylated tubulin immunostaining signal (Fig. S5G). Therefore, the GFP- Orbit was possibly distributed to axonemal microtubules emanating from the distal tips of basal body (Fig. S5B-D). Cp110 disappeared from centrioles at late spermatocyte stage, while Orbit continued to be localized on centrioles (Fig. S6C, D)."
We also added the following sentences explaining co-localization of Orbit with a transition zone marker, Chibby (line 178, page 10) and Fig. S3; "Next, the predominant localization at the distal end encouraged us to investigated whether the protein was accumulated to the transition zone (TZ) of centriole (Fig. S1B, Fig. S3). We induced expression of GFP-Orbit in the cells expressing a TZ protein with a fluorescence tag, Cby-Tomato. Cby was localized only with the most distal part of the Orbit-localizing region (Fig. S3C, C', C"). These observations suggest that Orbit over-expression

© 2021. Published by The Company of Biologists under the terms of the Creative Commons Attribution License (https://creativecommons.org/licenses/by/4.0/).

did not result in excess elongation of the TZ region."

3 In orbit overexpressing condition, the authors should check with different staining that the PACT stains the entire length of centriole and whether the axoneme and the transition zone are perturbed.
(Response)
We demonstrated that overexpression of Orbit resulted in excessive extension of centriole region including basal body and axonemal microtubules labelled by GFP-Orbit (Fig. 3A-D, I, Fig. S3B, C, E, and Fig. S5G).

Fine observations of centriole and basal body using super-resolution microscopy (SIM) reported that both PACT and Asl are localized along the whole basal body region (Jana et al., 2018). Our localization experiments of Asl and PACT using SIM revealed that Asl is localized through outer cylindrical centrioles, while PACT is distributed inside of whole centriole region except the proximal ends. In spite of a subtle differences in their distribution, overexpression of GFP-Orbit also resulted in excessive elongation of basal body region labelled by anti-Asl immunostaining (Fig. S4B). We added relevant sentences in Results (line 198-210, page 11-12). Therefore, we concluded that Orbit overexpression stimulated excessive elongation of basal body region of centrioles. Using these three centriole markers, PACT (Fig. 3I), Asl (Fig. S4B), and Orbit (Fig. S4A), we obtained the same conclusion that Orbit expression resulted in excess elongation of centrioles.
We also compared the localization of Orbit in centrioles to that of a transition zone protein. We added the following sentences in Results (line 178, page 10); "Next, the predominant localization at the distal end encouraged us to investigated whether the protein was accumulated to the transition zone (TZ) of centriole (Fig. S1B, Fig. S3). We induced expression of GFP-Orbit in the cells expressing a TZ protein with a fluorescence tag, Cby-Tomato. Cby was localized only with the most distal part of the Orbit-localizing region (Fig. S3C, C', C")."
Furthermore, we added the following sentences in line 211, page 12 and Fig. S3.

"In the cells over-expressing GFP-Orbit at the moderate level (*w; Cby-Tom/+;bam-Gal4/UAS-GFP-Orbit*), we sometimes observed centriole pairs with longer Orbit-localizing regions protruding from basal bodies (Fig. S6C, C"). In the cells, Cby was localized only with the most distal part, not the whole Orbit region. These observations suggest that Orbit over-expression did not result in excessive elongation of the TZ region. Moreover, we also observed the axonemal microtubules extended from basal bodies by anti- acetylated tubulin immunostaining (Fig. S5G). Consequently, overexpression of Orbit resulted in excessive elongation of axonemal microtubules extending from the basal bodies."

4 The authors should check for overstatement as they denote bias in the way the study was conducted. For example, the sentence: "the observation that Orbit localised around the distal plus end of centriole suggested that it was involved in centriole elongation". This observation might indeed indicate that or a role in the transition zone, or in anchorage of the centrioles or many other things. There are many occurrences of such overstatement.
(Response)

According to the reviewer's request and considering the reviewer 2's suggestion, we revised the sentence containing overstatement (line 184, page 10 in previous manuscript) as follows; (previous sentence)". These findings suggested that Orbit was possibly involved at the elongating end of the centriole in spermatocytes before meiosis."
(Revised) "These localization data, together with the known microtubule polymerization role of Orbit, suggested that it was possibly involved in elongation of the centriole at the distal end in spermatocytes before meiosis." (line 184, page 10).

(previous sentence) "These results clearly indicated that Orbit played an essential role in both microtubule polymerisation and centriole elongation in premeiotic spermatocytes." (line 208, page 1 in previous manuscript)

(Revised) " These genetic results suggest that Orbit plays an essential role in both microtubule polymerisation and centriole elongation in spermatocytes. "(line 226, page 12)

(previous sentence) "These results indicated that these two factors acted antagonistically for the

© 2021. Published by The Company of Biologists under the terms of the Creative Commons Attribution License (https://creativecommons.org/licenses/by/4.0/).

production of centrioles of specific length." (line 238, page 13 in previous manuscript)
(Revised)" These results suggest that these two factors act antagonistically for the production of centrioles of specific length."(line 260, page 14)

(previous sentence)" Klp10A, ,,,,, plays an indispensable role in regulation of centriole length (Delgehyr et al., 2012, this study), which must be determined by the balance between polymerisation and depolymerisation of the triplet microtubules."

(We revised the sentence thoroughly as follows)" Microtubule length can be determined by the balance between polymerisation and depolymerisation of a tubulin heterodimer into protofilament." (line 436, page 22)

5 I do not think it is reasonable in science to assume anything. So for the modulation of Orbit overexpression the authors should assess their statement either by quantitative immunofluorescence or by WB.
(Response)

According to the reviewer's request, we performed quantitative fluorescence of GFP-Orbit. We added the following a phrase to explain the quantitative method.
"By quantitative fluorescence that a total GFP-Orbit fluorescence were measured in each spermatocyte, " (line 200, page 11).

6 It would be interesting to know what happens if both klp10A and orbit are depleted to see whether the antagonism explain all the phenotype observed in klp10A mutant.
(Response)
We agree that it might be interesting to see what happens, if both *Klp10A* and *orbit* are depleted in spermatocytes or *klp10A* depletion in *orbit*^{7} spermatocytes, in order to confirm whether the antagonism hypothesis explains all the phenotype observed in *klp10A* mutant. In theory, experiments that see centriole phenotypes in both or either depletion conditions would be possible. However, we were wondering if interpretation of centriole phenotypes in the cells with double depletion seems complicated. It is not certain whether *Klp10* depletion and *Orbit* depletion could be performed at the same efficiency. Moreover, it is also uncertain whether the proteins work on centriole elongation in a manner of one-two-one correspondence between each other, as shown in regulation of cytoplasmic microtubules, We will address the issue in our future study.

7 There is no graph for the centriole length of CP110Delta, orbit overexpression.

(Response)

According to the reviewer's request, we added images and graph demonstrating a centriole length of *Cp110*$^{\Delta 1}$*/Y; bam>orbit* and some control cells in Fig. 6A-D and presented quantitative data in Fig. 6J, K. We also added relevant sentences describing the data (line 328-332, page 17-18 and line 338-341, page 18).

R1-8 The authors should present and attempt to understand the data on CP110 spindles. Indeed it is puzzling to think that they present "broken" centrioles (24%) that do not organise microtubules, on contrary of the other mutants.
(Response)

According to the reviewer's request, we added meiotic spindle images of primary spermatocytes in control (*bam-Gal4*/+) and *Cp110*$^{\Delta 1}$ mutant males as Fig. 8A and 8B, respectively. We also added several sentences describing about their spindle phenotypes in Results (line 397-399, 402-407, page 20), and the relevant Figure 8 legend (line 988, page 49).
We have found "broken" centrioles and extra full-length centrioles (overduplication) at a low frequency in *Cp110*$^{\Delta 1}$ spermatocytes (Fig. 6I). Consistently, we observed the null mutant cells having multipolar spindle microtubules at a low (10.4%) frequency among the meiosis I cells (Fig. 7C). In the multipolar spindle microtubules, most of centriole pieces labelled by mRFP-PACT was associated with spindle poles in the *Cp110* null mutant cells (Fig. 7B, B"). It may be puzzling that

© 2021. Published by The Company of Biologists under the terms of the Creative Commons Attribution License (https://creativecommons.org/licenses/by/4.0/).

the cells carrying abnormal centrioles were observed at a little higher frequency. Considering a previous result that 5% of the spermatocytes contained “over-duplicated” centrioles (Franz et al., 2013), we wonder if a total frequency (24%) of abnormal centrioles in *CP110*$^{□1}$ mutant males might be an over-estimation.

R1-9 The authors should be careful in their conclusions as some of their mutants are not null (hypomorphic or RNAi).
(Response)

Considering the reviewer’s concern, we added phrases in several sites of text for readers so as to recognize that we used hypomorphic orbit mutant, klp10A RNAi, and Cp110RNAi, instead of the null mutant.

Reviewer 2 Comments for the Author: Major points:

1. *Fig S1A - Do all centrioles marked with □-tubulin and PACT show a similar shift in signal? It would a be good to include a sentence about your hypothesis on why such a distribution is observed.*

(Response)

According to the reviewer’s comment, we added the following phrase enclosed in a parentheses in Results (line 161, page 9).
“(> 50 premeiotic spermatocytes from 10 male flies). “

We have the following hypothesis. However, to avoid exceeding the word limit, we decided not include these sentences in the text.
There is a cartwheel structure composed of Sas6 and Ana2 at the proximal ends. Our observation suggests that there may be a region or a separate structure between a pair of centrioles, on which the basal body microtubules are not associated, but the PACT is localized on the structure. Previous EM studies indicates that the cartwheel structure was present inside of cylindrical arrays of nine triplet microtubules (Lancarek and Bettencourt-Dias 2018, Jane et al., 2018). By contrast, fine EM observation of mammalian cells revealed other minute structures, a stalk and gamma-tubulin ring complexes between the proximal ends of two engaged centrioles (Firat-Karalar and Strearns, 2014). If PACT is distributed to either or both of these structures, we can interpret the differences in distribution between PACT and □-tubulin.
Alternatively, we cannot exclude that GFP-tubulin molecules consisting of the most proximal region of basal body microtubules might fail to emit GFP fluorescence due to some unknown reason such as some modification.

2. *Fig 2A/A’ - Orbit signal along the length of the centriole is not visible in the image provided. It appears as if Orbit is localised only in the region beyond the distal end of the centriole. An image like in Fig 2B will be appropriate.*

(Response)

According to the reviewer’s request, we replaced the Fig2A’ image for its longer exposed image so that our readers can recognize the localization signal more easily.

3. *Fig S2A - The graphs do not match the description provided in the figure legend. The bar graph of the control is missing. If centriole length was measured with GFP-Orbit signal, how were the control centrioles measured in the absence of a GFP-Orbit transgene?*

(Response)

Considering the reviewer’s concern, we added a bar chart of the control cells (*bam-Gal4*/+) in Fig. S4A (previous Fig. S2A) and added the following sentence in the relevant figure legend, as requested;
“The length in control cells was measured by anti-Asl immunostaining.” (Figure S4A legend)

4. *Despite centriole elongation, 100% of the centrioles were engaged in Orbit overexpression. For Klp10A RNAi, besides centriole elongation, were there any other phenotypes associated with defects in centriole integrity because of the long centrioles? If so, please do present the data in*

© 2021. Published by The Company of Biologists under the terms of the Creative Commons Attribution License (https://creativecommons.org/licenses/by/4.0/).

Figure 4.
(Response)

After reading the reviewer's comment, we realized the mistake in the Figure 5 legend. The control chart (grey bar) in Fig. 5G indicates to a frequency of centriole phenotype in *bam>dcr2 LacZ* , as written in line 847, page 42 in previous manuscript. We revised the mistake (line 947, page 47). We appreciate the reviewer's careful reviewing.
As shown in Fig. 7, we have observed disengaged pairs of centrioles and breakage of centrioles at a lower frequency even in spermatocytes overexpressing Orbit (7% (disengagement),and 7% (breakage)). Also, 9% and 8% of the cells from *bam>Klp10A* RNAi males contained disintegrated and disengaged centrioles, respectively. We presented representative images for breakage and disengagement in a spermatocyte of $Cp110^{D1}$*/Y;bam>Klp10ARNAi* as a novel finding in Figure 6E-G, instead of Fig.5 requested (previous Fig. 4), because Fig. 5 is demonstrated as evidences for alteration of centriole length. These phenotypes are consistent with a previous finding in Delgehyr et al., 2012.

5. *Fig 5A – Marker used for the centriole length measurements is not indicated in the figure legend.*
(Response)

According to the reviewer's request, we added the following sentence "The centrioles of the cells with each genotype were visualized by expression of mRFP-PACT and measured the length. " in the legend of Fig. 5A (line 927, page 46).

6. *Fig 5B-E – GFP-Orbit signal looks very elongated in these centrioles. It will be nice to have a brief description about this phenotype in the results section in addition to the centriole elongation and loss of centriole integrity phenotype – why does the signal appear like this/your hypothesis.*
(Response)

Simultaneous overexpression of *orbit* and depletion of *Klp10A* resulted in a production of overly long axonemal microtubules decorated by Orbit-GFP in spermatocytes (Fig. 5B-F). The microtubules extended from the distal end of centriole basal body labelled by mRFP-PACT. Orbit was distributed along centriole, from the basal body to CLR (Fig. 2B, Fig. S6). The protein is enriched on the distal ends of the axonemal microtubules. We added the following sentences explaining the Orbit localization in spermatocytes having both Orbit overexpression and Klp10A depletion in Results (line 253-256, page 14);
"Interestingly, spermatocytes harbouring Orbit overexpression and Klp10A depletion possessed an overly elongated GFP-Orbit signal protruding from the distal end of basal body. Orbit was distributed along centrioles overly extended from basal body and predominantly accumulated at the distal end of the centrioles (Fig. 2B, 5B)."
In addition, We added the following sentences in Discussion (line 456, page 23).

"Accordingly, we hypothesise that these are overly long axoneme microtubules produced as a consequence of excessively stimulated polymerization of tubulins by the overexpression. Orbit/CLASP has a microtubule binding activity to stimulate tubulin polymerization at the plus end (Inoue *et al.*, 2000, Al- Bassam *et al.*, 2010). Alternatively, we cannot exclude a possibility that Orbit polymerizes itself to construct microtubule-like structure on the distal tip of the basal body. It is interesting to investigate whether basal body and axonemal microtubules overly elongate in cells overexpressing Orbit without fluorescence tag."

7. *Lines 294-304 describe the phenotypes observed in CP110 mutants. However, the data has not been presented in the main/supplementary figures. Please include the data.*
(Response)

According to the reviewer's request, we added SIM images of centrioles in *Cp110*□ mutant spermatocytes as Fig. 6B and quantitative data of centriole length in Fig. 6J and K.

8. *For Figs 6A-H, for each of the genotypes, please briefly mention in the results section the effect on centriole length - whether it is shorter/longer than in the control in each scenerio.*

© 2021. Published by The Company of Biologists under the terms of the Creative Commons Attribution License (https://creativecommons.org/licenses/by/4.0/).

(Response)

According to the reviewer's request, we have measured the centriole length in SIM image files, and described the results briefly in Results (line 335, page 17).
"In the *Cp110* null mutant, average length of centrioles increased (1.20 □m on average, n=66 centrioles examined), compared to that in control spermatocytes (1.02 □m, n=53). Comparing to the length in the cells overexpressing Orbit (*bam>orbit*) (1.33 µm, n=49) or that in the *Cp110*$^{\Delta 1}$ mutant cells, the length significantly increased in *Cp110*$^{\Delta 1}$*/Y; bam>orbit* cells (Fig. 6A-D) (1.46 µm, n=21) (Fig. 6J, $p < 0.0001$ in both cases, Student's *t*-test). Consistently, the centrioles in *Cp110*$^{\Delta 1}$*/Y; bam>Klp10ARNAi* cells also significantly increased in length (Fig. 6G-I) (1.67 µm, n = 45, Fig. 6K), compared to that in the cells harbouring Klp10A depletion (*bam>Klp10ARNAi*) (1.48 µm, n = 52) (Fig. 6E-G) (Fig. 6K, $p < 0.0001$ in both cases). Contrary to the conditions that stimulate centriole elongation, the length did not change in the *Cp110*$^{\Delta 1}$ cells overexpressing Klp10A (*Cp110*$^{\Delta 1}$*/Y; bam>Klp10A* cells (0.94 µm, n=55), compared to that in *bam>Klp10A* cells (Fig. 6, H, I) (0.95 µm, n=51, Fig. 6K), even though centrioles decreased in length in both cases (Fig. 6K)."

9. For clarity, it will be better to briefly describe why no centriole integrity loss phenotypes were observed for Klp10A overexpression in CP110 mutant background and Klp10 overexpression spermatocytes.
(Response)
We have not observed the centriole integrity loss phenotypes in spermatocytes overexpressing Klp10A, even in CP110 null mutant background (Figure 6G-I). We currently consider that improvised construction of the basal body microtubules produced as consequence of Orbit overexpression and/or Klp10A depletion may be associated with the loss of centriole integration. We added the following hypothesis and describe our model in Discussion (line 515, page 26); "By contrast, the loss of centriole integrity phenotypes was not observed in the cells overexpressing a shortening factor, Klp10A. Hence, we consider that improvised construction of the basal body microtubules may be associated with the loss of integrity phenotype. And thereby, centriole engagement would be lost. We found centriole fragments with shorter diameter in the centriole microtubules. The presence of disintegrated centrioles supports this idea. Further investigations are necessary to test the current hypothesis."

10. Data for control and Cp110 mutant bipolar meiotic spindle is not shown in Fig 7. Please include the data.
(Response)

According to the reviewer's request, we added images of bipolar meiotic spindles in control and *Cp110* mutant cells in Fig. 8A and B, respectively. We also added sentences explaining meiotic spindle microtubules of these cells, and revised the numbers of abnormal cells and total cells examined (line 397- 399 and line 402-407, page 21).

Minor Points:

1. Fig 4F/F' - There appears to be a black box in the figure that is visible in the single DNA channel.

(Response)

We removed the black box as requested.

2. Line 233 - "Toward this purpose, we overexpressed Orbit and Klp10A simultaneously in.." 'Depleted' is missing in front of Klp10A.
(Response)

We revised the mistake as requested (line 251, page 13).

3. Fig 6B - PAC should be PACT.
(Response)

© 2021. Published by The Company of Biologists under the terms of the Creative Commons Attribution License (https://creativecommons.org/licenses/by/4.0/).

We revised the typo as requested (Fig. 7B) (previous Fig. 6B).

4. Line 277 – Citation should be (Galletta et al. 2016) instead of 2010.
(Response)
We removed this sentence to shorten the section.

5. *A recent study on Cep97 - 'Cep97 Is Required for Centriole Structural Integrity and Cilia Formation in Drosophila' (Dobbelaere et al. 2020) discusses its role in regulation of centriole length. It may be good to briefly discuss the centriole elongation findings of the Cep97 paper in the Discussion section to complement the findings of CP110 presented in this paper.*

(Response)

To complement the findings of CP110 presented in this study, we also examined a centriole phenotype in *Cep9*^{LL} mutant, which is a homozygote for a hypomorphic allele. We have observed precocious disengagement of centriole sets in every mutant spermatocytes before meiosis, as described in the original manuscript. In contrast, Dobbelaere and colleagues have recently reported a different phenotype in a null mutant for the gene (Dobbelaere et al. 2020). The authors did not described any other phenotypes than excessive elongation of centrioles. We observed the same phenotype in the mutant cells hemizygous over a deficiency uncovering the gene. Rather, the hemizygoous cells showed more sever phenotypes. To clarify the differences in these phenotypes, we need more experiments. To avoid exceed of a word limit of the journal, we decided to move all data regarding Cep97 to our next manuscript.

Reviewer 3 Comments for the Author:

line 32: "physically" - the only evidence for physical interaction is the proximity ligation assay which "suggest" but does not convincingly prove a physical interaction. Someone would expect here in-vitro binding assays, Co-IP-s to this end. Therefore I suggest removing this word.
(Response)

According to the reviewer's suggestion, we removed the word "physically" and revised the sentence as follows (line 32-33, page 3):
"Furthermore, Cp110 in the distal tip complex was closely associated with factors involved in centriolar dynamics at the distal end."

line 55: sentence "The C-terminal domain of Dplp (a PCM protein), called the pericentrin-AKAP450 centrosomal targeting domain (PACT), also localises along the centrioles." These sentence in the introduction break the nice line of thought of the text. I understand that the authors wish to highlight PACT since they will use this later as a marker based on which they measure the centriole length. I suggest to put this reference sentence in the result section after line 160.
(Response)

As suggested, we moved the relevant sentence after line 156, page 9 (line 160 in previous manuscript),.

line 175: just the localization on its own doesn't raise the possibility. I suggest rephrasing: "localization together with the known Mt polymerization role suggest..."
(Response)

According to the reviewer's suggestion, we rephrased the sentence (line 184, page 10).

line 206: please indicate in a half-sentence you used hypomorph orbit allele (a reader interested in Drosophila genetics might want to know why only this)? Was null not available? Is the orbit null lethal in early developmental stage? Using hypomorph raises the possibility that we don't see the maximum of the effect of orbit loss, but it is not a problem since still demonstrate the function.

© 2021. Published by The Company of Biologists under the terms of the Creative Commons Attribution License (https://creativecommons.org/licenses/by/4.0/).

I'm just curious if it is known the phenotype of an orbit null. And if yes, would be nice to mention here.
(Response)

We used an hypomorphic allele, *orbit*^{7} of *orbit* gene in this experiment, as we failed to examine spermatocytes in homozygotes for null alleles, because of their earlier death. According the reviewer's suggestion, we added the following sentence in line 222, page 12;
"Spermatocytes failed to be observed in *orbit* mutants homozygous for the null allele because of their earlier stage death."
However, it is interesting to examine whether a similar centriole phenotype appears in larval neuroblasts from the null mutants using EM or super resolution microscope. We appreciate the reviewer's comment. We will include the experiment in our next study.

line 218: very good that authors coexpressed dicer2 since the adults fly doesn't express well Dicer2 and that diminishes the efficiency of RNAi thus dcr2 coexpression is needed. A reader with little fly genetics background might wonder what does UAS-dcr2 mean, so it would be great to mention in a sentence in the results or in the figure legend and explain well in Materials and Methods.
(Response)

According to the reviewer's request, we revised the relevant sentence as follows;

"When we induced dsRNA against each mRNA, we also induced co-expression of the *dsr2* mRNA encoding a Dicer2 double-stranded RNA-specific endonuclease in every RNAi experiment to raise the RNAi efficiency in testis cells, where the *dcr2* expresses at a lower level (flybase; https://flybase.org/reports/FBgn0034246) in testis cells." (line 605, page 30).

line 233: Please correct this sentence. It is obvious from the given genotype that it is not simultaneous overexpression of Orbit and Klp10A but overexpression of orbit combined with Klp10A knockdown.

(Response)

We revised the mistake as requested (line 251, page 13).

There is a typo on Figure 6 C and D, "dcr2" is mistyped as "dsr2".

(Response)

We removed the word "dcr2" from Fig. 6 to make the figure easier to see.

Second decision letter

MS ID#: JOCES/2020/251231

MS TITLE: Orbit/CLASP determines centriole length by antagonising Klp10A in *Drosophila* spermatocytes

AUTHORS: Tsuyoshi Shoda, Kanta Yamazoe, Yuri Tanaka, Yuki Asano, and Yoshihiro H. Inoue
ARTICLE TYPE: Research Article

I am happy to tell you that your manuscript has been accepted for publication in Journal of Cell Science, pending standard ethics checks.

© 2021. Published by The Company of Biologists under the terms of the Creative Commons Attribution License (https://creativecommons.org/licenses/by/4.0/).

Reviewer 2

Advance summary and potential significance to field

The mechanism of centriole elongation in Drosophila remains elusive with only a few studies that have attempted to address this question. Studies of Klp10A have established that it is an important regulator of centriole length in Drosophila.
Given that centriole length is believed to be regulated as a balance between polymerisation and depolymerisation of centriolar microtubules, this manuscript's focus on Orbit's role as a polymerisation factor complements studies of Klp10A. Therefore, the manuscript would be of interest to the field and I support its publication.
The revision of the paper from Shoda and colleagues has addressed the queries and suggestions that I raised in my previous review of the submission. I have no further comments.

Comments for the author

The revision of the paper from Shoda and colleagues has addressed the queries and suggestions that I raised in my previous review of the submission. I have no further comments.

Reviewer 3

Advance summary and potential significance to field

In this manuscript the authors used the versatility of Drosophila genetics and cell biology to demonstrate that the microtubule polymerizing factor Orbit/CLASP contributes to the determination of centriole length and antagonizes Klp10A. Moreover, the authors bring into the picture the CP110 and the distal tip complex and also used the very reliable genetic interactions to demonstrate how this complex interacts with Orbit and Klp10A to ensure proper centriole lenght. In addition to the significance of these results giving to hot centriole elongation field, it also has relevance in cancer research demonstrating that centriole over-elongation by disturbance of these factors leads to loss of centriole integrity, consequently multipolar spindle thus chromosomal instability.
This reviewer does encourage the acceptance and publication of this paper.

Comments for the author

Thank you for considering and addressing my comments. I do encourage the acceptance of the revised manuscript.

© 2021. Published by The Company of Biologists under the terms of the Creative Commons Attribution License (https://creativecommons.org/licenses/by/4.0/).
